# Multivariate metabotyping of plasma predicts survival in patients with decompensated cirrhosis

**DOI:** 10.1016/j.jhep.2016.01.003

**Published:** 2016-05

**Authors:** Mark J.W. McPhail, Debbie L. Shawcross, Matthew R. Lewis, Iona Coltart, Elizabeth J. Want, Charalambos G. Antoniades, Kiril Veselkov, Evangelos Triantafyllou, Vishal Patel, Oltin Pop, Maria Gomez-Romero, Michael Kyriakides, Rabiya Zia, Robin D. Abeles, Mary M.E. Crossey, Wayel Jassem, John O’Grady, Nigel Heaton, Georg Auzinger, William Bernal, Alberto Quaglia, Muireann Coen, Jeremy K. Nicholson, Julia A. Wendon, Elaine Holmes, Simon D. Taylor-Robinson

**Affiliations:** 1 Division of Digestive Health, Department of Surgery and Cancer, Faculty of Medicine, Imperial College London, 10th Floor QEQM Wing, St Mary’s Hospital Campus, South Wharf Street, London NW1 2NY, United Kingdom; 2 Institute of Liver Studies, King’s College Hospital, Denmark Hill, London SE19 2RS, United Kingdom; 3 Computational and Systems Medicine, Department of Surgery and Cancer, Faculty of Medicine, Imperial College London, Sir Alexander Fleming Building, Exhibition Road, South Kensington, London SW7 2AZ, United Kingdom

**Keywords:** ALF, acute liver failure, ACLF, acute on chronic liver failure, CLIF-SOFA, chronic liver failure sequential organ failure assessment, CPS, Child-Pugh Score, CPMG, Carr-Purcell-Meiboom-Gill, CV-ANOVA, cross-validated analysis of variance, ESI, electrospray ionisation, GC-MS, gas chromatography mass spectrometry, INR, international normalised ratio, LPC, lysophosphatidylcholine, MELD, model for end-stage liver disease, MHE, minimal hepatic encephalopathy, MS, mass spectrometry, NMR, nuclear magnetic resonance, PC, phosphocholine, PCA, principal components analysis, PLSDA, partial least squares discriminant analysis, OPLS, orthogonal projection least squares, STOCSY, statistical correlation spectroscopy, TOF, time-of-flight, UPLC, ultra-performance liquid chromatography, UKELD, United Kingdom end-stage liver disease, Metabonomics, Metabolomics, Metabolic profiling, Personalised medicine, Outcome prediction, Acute on chronic liver failure

## Abstract

**Background & Aims:**

Predicting survival in decompensated cirrhosis (DC) is important in decision making for liver transplantation and resource allocation. We investigated whether high-resolution metabolic profiling can determine a metabolic phenotype associated with 90-day survival.

**Methods:**

Two hundred and forty-eight subjects underwent plasma metabotyping by ^1^H nuclear magnetic resonance (NMR) spectroscopy and reversed-phase ultra-performance liquid chromatography coupled to time-of-flight mass spectrometry (UPLC-TOF-MS; DC: 80-derivation set, 101-validation; stable cirrhosis (CLD) 20 and 47 healthy controls (HC)).

**Results:**

^1^H NMR metabotyping accurately discriminated between surviving and non-surviving patients with DC. The NMR plasma profiles of non-survivors were attributed to reduced phosphatidylcholines and lipid resonances, with increased lactate, tyrosine, methionine and phenylalanine signal intensities. This was confirmed on external validation (area under the receiver operating curve [AUROC] = 0.96 (95% CI 0.90–1.00, sensitivity 98%, specificity 89%). UPLC-TOF-MS confirmed that lysophosphatidylcholines and phosphatidylcholines [LPC/PC] were downregulated in non-survivors (UPLC-TOF-MS profiles AUROC of 0.94 (95% CI 0.89–0.98, sensitivity 100%, specificity 85% [positive ion detection])). LPC concentrations negatively correlated with circulating markers of cell death (M30 and M65) levels in DC. Histological examination of liver tissue from DC patients confirmed increased hepatocyte cell death compared to controls. Cross liver sampling at time of liver transplantation demonstrated that hepatic endothelial beds are a source of increased circulating total cytokeratin-18 in DC.

**Conclusion:**

Plasma metabotyping accurately predicts mortality in DC. LPC and amino acid dysregulation is associated with increased mortality and severity of disease reflecting hepatocyte cell death.

## Graphical abstract

**Figure:**
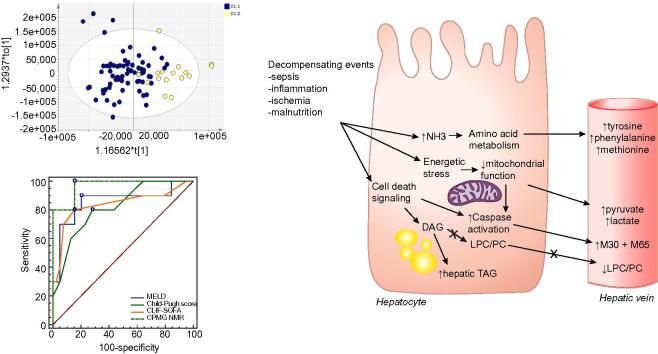

## Introduction

The global incidence of cirrhosis is rising rapidly, owing to an increased prevalence of alcohol-related liver disease, non-alcoholic fatty liver disease, and viral hepatitis [1]. Patients with cirrhosis are prone to decompensation, requiring hospital treatment and can progress to acute on chronic liver failure (ACLF) [2], which requires admission to intensive care with an associated high short-term mortality and significant economic cost [3].

Several methods of outcome prediction in cirrhosis are currently used. The Model for End-Stage Liver Disease (MELD) is the most commonly applied and is used for listing and prioritisation in liver transplantation throughout the world. Despite the success of MELD, several limitations exist concerning the reproducibility of prothrombin time measurement and the limitations of creatinine [4] as a marker of renal function in patients with cirrhosis. The performance of MELD for outcome prediction is best in patients with stable cirrhosis, but is less accurate for patients with acute on chronic liver failure (ACLF) [5]. Therefore organ failure based scores such as the recently developed CLIF-SOFA (chronic liver failure sequential organ failure assessment) score have been developed [2], [6].

Exploratory metabolic profiling or metabotyping involves untargeted measurements of low molecular weight (<1 kDa) compounds using nuclear magnetic resonance (NMR) spectroscopy or mass spectrometry (MS) in biofluids or tissues [7]. The response of complex spectral data is then assessed using multivariate statistical techniques [8] to determine which metabolites or metabolite combinations most accurately describe differences seen between classes (control or diseased cohorts or a state pre- or post-intervention (e.g. drug treatment)).

Since the metabolic profile is typically comprised of hundreds or thousands of signals depending on the technique, it is, potentially, a highly valuable methodology in delivering personalised healthcare [9], namely a highly discriminant prediction of response or diagnostic accuracy. The use of pre-interventional phenotypes to predict the outcome of intervention based on mathematical modelling is termed “pharmacometabonomics” [10] and the approach has been widely used to predict drug metabolism [11] and outcomes of cancer therapy, toxicity [12] and safety.

Metabolic profiling has been applied in inflammatory bowel disease [13], [14], hepatocellular carcinoma [15], [16] and to a limited extent, liver failure [17]. The metabolites detected by NMR reflect a number of roles of the liver in glucose, lipid, amino acid and urea metabolism and these have been investigated in acute liver failure (ALF) by proof-of-principle measurements [17], [18] and metabolic phenotypes specific to patients with minimal hepatic encephalopathy (MHE) [19] or high MELD score [20].

Liver failure secondary to hepatitis B has been characterised using MS in tandem with either gas chromatography (GC-MS) or liquid chromatography (LC-MS) [21]. Decreases in plasma glycerophosphocholine and phosphatidylcholine (PC) levels occur [22], and are common markers between different aetiologies of cirrhosis [23], [24]. Apoptosis of hepatic endothelial beds may be responsible for this lipid dysregulation. Higher levels of circulating cell death markers have been demonstrated in ALF [25], [26], and recently in decompensated cirrhosis (DC) and ACLF [27]. No studies have assessed how profiling could prognosticate and none uses a combination of technologies to develop a global overview of the metabolic signature of poor survival in DC.

In this study, we metabotype plasma in patients with DC to: 1) determine and validate a ^1^H NMR metabotype of 90-day mortality, which we hypothesise could be more accurate than MELD or CLIF-SOFA; and 2) characterise the lipids of the ^1^H NMR profile by UPLC-TOF-MS.

## Materials and methods

### Patients and sample collection

Between December 2008 and January 2011, 80 patients with DC referred to the Institute of Liver Studies, Kings College Hospital, London, were recruited and followed for 90 days. Cirrhosis was defined by at least two compatible diagnostic tests from the following: liver biopsy (fibrosis grade 5 or more), radiologic (ultrasound, computed tomography or magnetic resonance imaging), clinical (presence of hepatocellular jaundice/ascites/hepatic encephalopathy [HE]) or biochemical (hyperbilirubinaemia, prolonged prothrombin time and/or thrombocytopaenia) to provide evidence of cirrhosis. Outcome was defined as either spontaneous survival or death/transplantation. Here, we define decompensation as an acute episode of variceal bleeding, jaundice, encephalopathy, ascites, sepsis or renal dysfunction requiring hospital admission. The study was approved by the local ethics committee (#08/H0702/74) and patients, or their nominee gave written, informed consent within 48 h of presentation. Exclusion criteria were presence of hepatocellular carcinoma, non-hepatic malignancy, non-cirrhotic portal hypertension, chronic liver disease but not cirrhotic and acute liver failure. Liver intensive care unit admission was offered to patients if deemed appropriate by the referring hepatologist and/or intensivist. All patients were managed following standard evidence-based protocols by a specialist multidisciplinary team [28], [29].

Blood was drawn within 24 h of admission to hospital into lithium heparin-containing vacuum tubes (BD Vacutainer, BD, Franklin Lakes, NJ, USA) and centrifuged at 12,000 g for 10 min within 1 h of sample collection. Plasma aliquots were stored immediately at −80 °C until further analysis. Data collected at time of sample collection were age, gender, aetiology of cirrhosis, past medical history, medication use, dietary history and 72 h dietary recall, alcohol and recreational drug use, recent exercise history, bedside physiology and blood biochemistry. The Child-Pugh Score (CPS) [30], MELD [31], United Kingdom end-stage liver disease (UKELD) [32], CLIF-SOFA [2], CLIF AD [33] and CLIF-C ACLF [6] scores were calculated from data taken on the same day as blood was drawn for metabolic profiling. Twenty patients with stable cirrhosis and 20 age- and sex-matched healthy controls (HC) with no history of liver disease, excess alcohol or recreational drug use, or hepatotoxic or over the counter medication usage were also enrolled following written, informed consent (REC #09/H0712/82). From December 2011 to January 2015 a further validation cohort of 101 patients (in two cohorts of 59 and 42 respectively) hospitalized with DC and 27 HC were recruited. The primary outcome was 90 day survival.

### NMR data acquisition and processing

Plasma aliquots were thawed to room temperature. Aliquots of 200 μl plasma were added to 400 μl of a solution containing 0.9% NaCl and D_2_0 as previously described [34]. This was centrifuged at 12,000 g for 10 min and 550 μl of supernatant placed into 5 mm NMR tubes (Norell, Landisville, NJ, USA). Data were acquired in a random, blinded order on a Bruker Avance™ spectrometer (Bruker GmbH, Rheinstetten, Germany) with a 5 mm TXI probe operating at 600.13 MHz and 300 K. Data were acquired using a 1D technique using a standard pulse sequence with pre-saturation of the water resonance and Carr-Purcell-Meiboom-Gill (CPMG) spin echo sequences to attenuate the broader peaks arising from lipids and proteins, as described in Supplementary methods along with pre-processing techniques.

### UPLC-TOF-MS data acquisition and processing

Plasma aliquots were thawed to room temperature. 50 μl of plasma were added to 150 μl of ice cold 100% methanol, vortexed briefly, and kept at −20 °C for 20 min. Following 10 min of centrifugation at 12,000 g, 170 μl of supernatant was transferred to Eppendorf™ containers (Eppendorf, Stevenage, UK). The supernatants were dried during 90 min of centrifugation under vacuum at 40 °C (Savant SpeedVac, ThermoScientific, Asheville, NC, USA). Dry material was suspended in 120 μl of high purity water (UpS grade, Romil Ltd, Cambridge, UK) and sonicated for 30 min. One-hundred μl of each sample was added in a random order in a 96-well plate while 20 μl were reserved from each to make a quality control (QC) sample. Samples were then transferred to the sample manager of a Waters Acquity UPLC system (Waters Corporation, Milford, MA, USA) maintained at 4 °C. Reversed-phase chromatography was conducted using a gradient from acidified water to acidified methanol as detailed with MS conditions in Supplementary material. Extraction of features detected by mass spectrometry across the entire sample set was performed using XCMS software [35] operating in the R computing environment. A data matrix of samples analysed *vs.* detected features and corresponding intensity values was produced and analysed by multivariate analysis using SIMCA P software (v 12.0.1 Umetrics AB, Umeå, Sweden).

### Multivariate analysis of the plasma spectral profiles

For both NMR and UPLC-TOF-MS data, principal components analysis (PCA) was performed to visualise any inherent clustering and identify outliers. Orthogonal projection to latent structure (OPLS) analysis was performed to maximise class differences while minimising variability unrelated to class. The R^2^ value was calculated to give a measure of the goodness-of-fit or amount of variability explained by the model. A cross-validated Q^2^ statistic (leave-one-out algorithm) was calculated as a quantitative measure of the predictability of the model for the Y variable. The cross-validated-analysis-of-variance (CV-ANOVA) statistic corresponds to a null hypothesis of equal predictive residuals between the models under investigation (*p* <0.05 suggests the model is superior to one chosen at random). Sensitivity and specificity were calculated from the cross-validated Y-predicted variable. The S-line and S-plot loadings (displaying correlation *vs.* covariance of spectral variables) was used to determine the metabolites contributing to class separation for NMR and UPLC-MS data respectively. AUROC comparison was by the Hanley-McNeill method. Univariate comparison of plasma levels of metabolites was performed by one way ANOVA within MedCalc v12.1.4 (MedCalc Software, Mariakerke, Belgium).

### Measurements of cell death activity

Plasma levels of total (un-cleaved) cytokeratin-18 (M65) and caspase cleaved cytokeratin-18 (M30) were quantified with commercially available ELISAs (Peviva AB, Bromma Sweden), previously validated in clinical trials and used according to manufacturer’s instructions as described elsewhere [36]. Levels were taken in patients with DC, stable chronic liver disease (CLD) and HC. To determine the role of the liver in cell death activity, M30 and M65 levels were also measured in seven patients with DC at the time of liver transplantation prior to hepatectomy.

### Liver samples and Immunohistochemistry

Explant liver tissue was available in six patients undergoing orthotopic liver transplantation for DC. Background liver from liver resection specimens containing metastatic adenocarcinoma from colorectal primaries (n = 4) served as normal control tissue. Liver tissue from a patient with post-liver transplant recurrent HCV infection was used as a positive control. Negative controls were obtained by omitting the primary antibody. Tissue samples were taken for diagnostic histological examination and formalin fixed, paraffin-embedded (FFPE), and stained with H&E using a standard procedure. FFPE tissue was cut at 4 μm and serial sections were picked up on poly-l-lysine coated slides, which were manually stained using the M30 CytoDEATH primary antibody (product number 12140322001, Roche, UK; dilution 1:10).

M30 enzymatic immunohistochemistry are described in the Supplementary methods.

## Results

Eighty patients (median age 55 (23–75) years, 60 male) comprised the derivation cohort (Table 1). Sixty-two patients spontaneously survived. In keeping with other reports of outcome in these groups, non-survivors exhibited a higher MELD, UKELD and CPS and were associated with higher levels of requirements for organ support therapies. Aetiology was not associated with mortality. Thirty-seven patients met criteria for ACLF at the time of recruitment (15 patients with ACLF grade 1, 17 with ACLF grade 2 and 5 with ACLF grade 3). The remainder (43) did not meet CANONIC criteria for ACLF.

### NMR spectroscopic multivariate analysis

#### Comparisons between controls and patients with cirrhosis

Examples of ^1^H NMR spectral data for plasma are shown in Supplementary Fig. 1. Clear differences can be seen in the glucose, lipid and lactate resonances between controls and patients with liver disease. PCA comparing controls with liver disease patients in a 3 component model gave an R^2^X statistic of 0.75 with a Q^2^Y of 0.54 (Supplementary Fig. 2). OPLS-DA analysis gave similar robust differentiation of the NMR spectral profile (R^2^X = 0.67, R^2^Y = 0.75, Q^2^Y = 0.59, CV-ANOVA 10^−23^, sensitivity 97%, specificity 100%). Resonances increased in patients with cirrhosis were lactate, glucose, methionine and pyruvate, while those reduced in patients with cirrhosis were lipid, choline and phosphocholine resonances (see Table 2; Supplementary material). OPSLDA modelling accurately discriminated patients with and without ACLF profile (R^2^X = 0.25, R^2^Y = 0.49, Q^2^Y = 0.38, CV-ANOVA 10^−9^, sensitivity 83%, specificity 90%) but did not discriminate by aetiology.

#### Outcome prediction in patients with DC

**Fig. 1:**
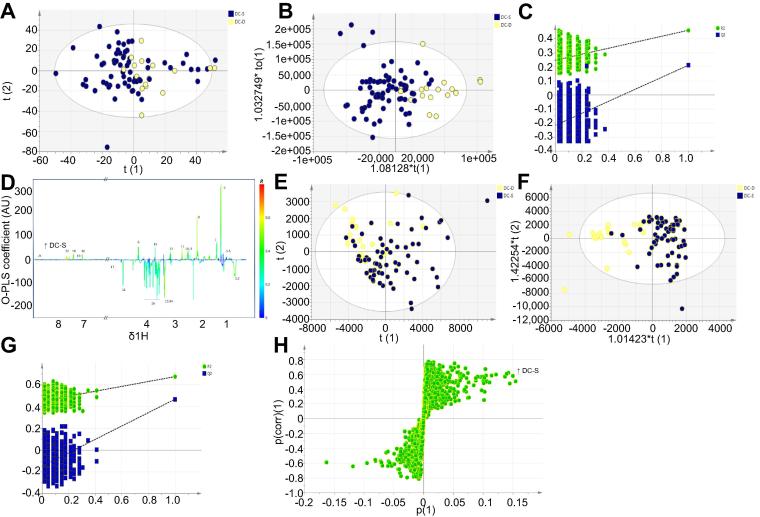
^**1**^**H NMR and UPLC-MS plasma profile models.** These multivariate models demonstrate discrimination of patients with DC who survive or die (A–D) ^1^H NMR data. (A) Principal components analysis (PCA) scores plot 3 component model R^2^X = 0.75 Q^2^ = 0.54. (B) Orthogonal projection least squares discriminant analysis (OPLS-DA) scores plot (1 + 2 + 0) R^2^X = 0.57 R^2^Y = 0.46 Q^2^ = 0.25. (C) permutation analysis (D) S-line loading plot (E–H) UPLC-MS positive mode data (E) PCA scores plot (3 components) R^2^X = 0.54 Q^2^ = 0.42 (F) OPLS-DA scores plot (1 + 2 + 0) R^2^X = 0.52 R^2^Y = 0.67 Q^2^ = 0.42 (G) permutation analysis (H) S-plot loadings. ^1^H NMR peak annotations are as per Table 2. (This figure appears in colour on the web.)

When 90-day survival was used as the class variable within the cohort of patients with DC, a discriminant model was produced (3 component OPLS-DA (R^2^X = 0.57, R^2^Y = 0.46, Q^2^Y = 0.39) with 100% sensitivity and 85%, CV-ANOVA 10^−6^; Fig. 1). Higher levels of lactate, tyrosine, methionine and phenylalanine were found in patients with poor outcome, whereas lower levels of the lipid resonances (VLDL, LDL) and particularly the choline/phosphocholine resonances at δ 3.22 were demonstrated. The resonance at δ 3.22 contains peaks attributable to both choline and the associated phosphocholine macromolecule and therefore reversed-phase UPLC-TOF-MS was performed for detailed metabolite characterisation (see below).

#### External validation of NMR profiling as predictive of hospital survival

**Fig. 2:**
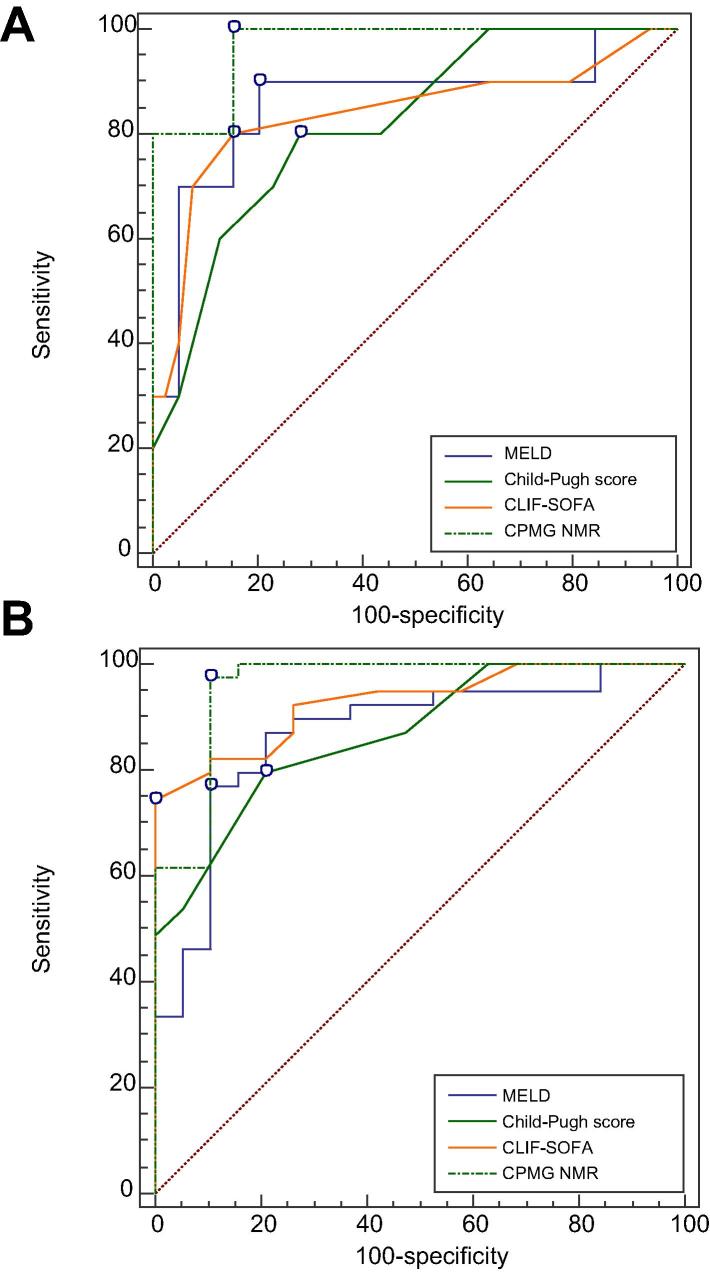
**Comparison of outcome prediction performance by Area Under Receiver Operating Curve (AUROC) analysis.** AUROC comparisons of CPMG (Carr-Purcell-Meiboom-Gill), NMR (nuclear magnetic resonance), MELD (model for end-stage liver disease), CLIF-SOFA (chronic liver failure sequential organ failure assessment), UPLC-MS (ultra-performance liquid chromatography mass spectrometry). (A) Derivation Cohort CPMG NMR profile (AUROC 0.95 (0.90–1), sensitivity 100%, specificity 79%, *p* <0.001); CLIF-SOFA – AUROC 0.87 (0.77–0.93) sensitivity 78%, specificity 91%, *p* <0.001); MELD – AUROC 0.81 (0.66–0.96), sensitivity 78%, specificity 86%, *p* <0.001); Child-Pugh Score – AUROC 0.87 (0.76–0.98), sensitivity 83%, specificity 78%, *p* <0.001. (B) Validation Cohort CPMG NMR profile (AUROC 0.96 (0.90–1), sensitivity 98%, specificity 84%, *p* <0.001); CLIF-SOFA – AUROC 0.93 (0.86–0.99) sensitivity 74%, specificity 100%, *p* <0.001); MELD – AUROC 0.87 (0.66–0.96), sensitivity 89%, specificity 79%, *p* <0.001); Child-Pugh Score – AUROC 0.87 (0.76–0.98), sensitivity 80%, specificity 79%, *p* <0.001. (This figure appears in colour on the web.)

In a separate cohort of 59 patients with DC (median age 56 (43–65), 54% male, 14D; 45 SS, see Supplementary material) the 3 component NMR profile developed on the training set was used to predict outcome in this fully external validation cohort. The outcome prediction accuracy was again excellent with a sensitivity of 98% (87–100%), specificity 89% (67–99%), positive likelihood ratio 9 (3–34), negative likelihood ratio 0.03 (0.004–0.2), AUROC 0.96 (0.90–1.00) (Fig. 2). In a further validation cohort both the differences between patients with cirrhosis (n = 42) and controls (n = 27) and discriminatory ability for predicting 90-day mortality were confirmed (see Supplementary data).

By limiting the metabolites used for prediction to those with a variable importance of more than unity, a simplified metabolite array of glycerophopshocholine (GPC), lactate, LDL, phenylalanine, methionine, pyruvate, glucose and tyrosine, retained a discriminatory accuracy (R^2^X = 0.61 R^2^Y = 0.41 Q^2^Y = 0.15, CV-ANOVA *p* = 0.02, AUROC 0.86, sensitivity 92%, specificity 85%).

#### Characterisation of lipids by UPLC-TOF-MS multivariate analysis

In the dataset resulting from positive ion formation (examples in Fig. 2), accurate discrimination between patients with cirrhosis and healthy controls was attained with PCA (3 component model R^2^X = 0.53, Q^2^Y = 0.51) and confirmed on subsequent OPLS-DA (3 component model – R^2^X = 0.52, R^2^Y = 0.67, Q^2^Y = 0.42, sensitivity 85%, specificity 97%, CV-ANOVA 3 × 10^−14^).

A two component model was used for discrimination between survivors and non-surviving patients with decompensated cirrhosis, producing a valid model for discrimination of survival (Fig. 1; R^2^X = 0.47, R^2^Y = 0.54, Q^2^Y = 0.40, AUROC 0.94, sensitivity 100%, specificity 85%, CV-ANOVA *p* = 10^−8^). Major discriminatory metabolites are listed in Supplementary Table 1. A collection of masses related to adducts of other lysophosphatidylcholines (LPCs) and phosphatidylocholines (PC) were identified as being reduced in non-surviving patients as shown in Table 3, confirming the importance of downregulation of a number of LPC/PC species in increasing severity of liver disease and in outcome prediction. See Supplementary results for negative mode ionisation.

#### Cell death markers increased in DC and correlate with LPC concentrations

**Fig. 3:**
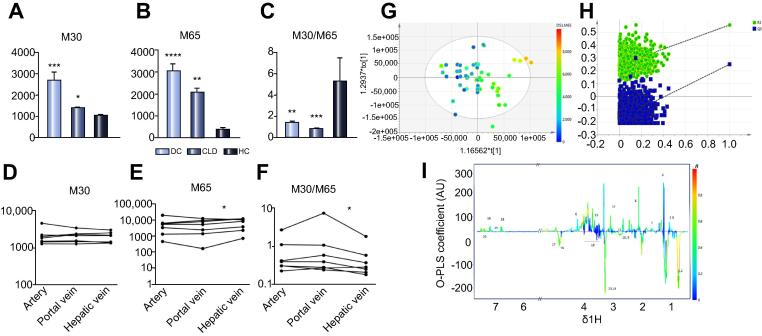
**Analysis of markers of cell death in peripheral blood, across the liver and in correlation with metabolic profiling.** (A–C) Comparison of markers of cell death in peripheral blood on day 1. DC, Decompensated cirrhosis; CLD, chronic stable liver disease; HC, healthy control. Kruskall Wallis test with Dunns test for multiple comparisons (∗*p* <0.05, ∗∗*p* <0.01, ∗∗∗*p* <0.001, ∗∗∗∗*p* <0.0001) data are expressed as mean(SEM). (D–F) Total and caspase cleaved cytokeratin-18 levels measured by ELISA from samples taken from the hepatic vein (HV), portal vein (PV) and systemic artery (ART) at the time of liver transplant prior to hepatectomy in n = 7 patients. (D) M30 levels demonstrating no gradient (E) M65 levels demonstrating positive gradient between portal and hepatic vein (E) M30/M65 ratio demonstrating negative ratio between portal and hepatic veins. All M30 and M65 levels are in IU/L (logarithmic scale for D-F, Paired *t* test n.s., non-significant ∗*p* <0.05). (G-I) OPLS modelling of ^1^H NMR plasma of patients with DC and M65 levels (G) OPLS model (1 + 1 + 0 components, R^2^X = 0.39 R^2^Y = 0.56 Q^2^ = 0.27 CV-ANOVA *p* = 0.004) (H) permutation test demonstrating validity of model (I) OPLS S-line loading plot demonstrating correlation with M65 levels and ^1^H NMR measured metabolites in particular LDL, PC (negative correlation), alanine, methionine, phenylalanine and tyrosine (positive correlation; peak annotations as for Fig. 1 and Table 2). (This figure appears in colour on the web.)

Levels of M30 were elevated in both CLD and DC compared with HC (Fig. 3). Similarly, the plasma levels of M65 were also highest in DC patients, compared to CLD and HC. M30/M65 ratios were lowest in DC, compared to other groups. M30 and M65 were correlated with MELD score (M30 *vs.* MELD r = 0.40, *p* = 0.002; M65 *vs.* MELD r = 0.39, *p* = 0.004). On OPLS modelling M65 levels correlated negatively with the LDL (pcorr −0.85) and PC (pcorr −0.74) resonances on ^1^H NMR and correlated positively with alanine (0.51), methionine (0.49), tyrosine (0.66) and phenylalanine (0.74, *p* <0.001 for all correlations model CV-ANOVA *p* = 0.004). M30 and the apoptotic index M30/M65 did not produce valid OPLS models with the NMR profile.

Of the identified lipids on UPLC-MS, LPC (16:0) was negatively correlated with M30 (r = −0.30, *p* = 0.010) and M65 (r = −0.39, *p* = 0.002) levels and also predicted hospital survival (AUROC 0.76 (95% CI 0.66–0.84), *p* <0.001).

M65 levels performed less well at outcome prediction (AUROC 0.66 (95% CI 0.54–0.76) *p* = 0.02). M30 and M30/M65 ratio were not independently associated with outcome.

#### Cross liver sampling demonstrates hepatic endothelial beds as source of total cytokeratin-18 in DC

**Fig. 4:**
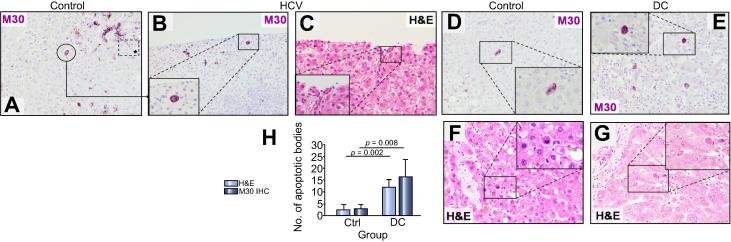
**Histological analysis of liver tissue.** These demonstrate apoptosis in explanted liver tissue from control and DC cases. (A) Representative micrograph showing a homogeneous cytoplasmic pattern in a cell morphologically similar to acidophilic bodies from H&E stains, in explanted control liver tissue (∗ intense coarse granular cytoplasmic staining pattern of hepatocytes regarded as non-specific staining and excluded from the quantitative assessment). (B) Representative micrograph demonstrating homogeneous cytoplasmic staining for M30 in cells with morphologic features suggestive of apoptotic bodies (C), on a biopsy from a patient with post-transplant recurrent HCV infection. (D–G) Representative micrographs of M30^+^ apoptotic cells and acidophilic bodies on H&E stain in the control and DC groups showing an increased numbers in the latter. (H) Enumeration of apoptotic cells in control (n = 4) and DC groups (n = 6), as assessed by enzymatic immunohistochemistry and H&E staining. (This figure appears in colour on the web.)

Blood sampling from the systemic artery, portal and hepatic veins at the time of liver transplantation prior to hepatectomy demonstrated higher levels of M65 in the hepatic vein compared to portal vein (*p* = 0.047) and lower M30/M65 ratios in the hepatic vein, compared to portal vein (*p* = 0.027, see Fig. 4).

The quantitative analysis of apoptotic cells in the control and DC groups on the M30-stained slides revealed a significant increase in apoptotic cell numbers in the DC group compared to controls (*p* = 0.008). This finding was supported by a similar increase as assessed on the H&E stained slides from the same cases (see Fig. 4, *p* = 0.002).

#### Comparison of outcome prediction abilities of profiling techniques

The AUROC curves analysis of the metabolic profiling techniques in comparison with accepted clinical decision making tools is shown in Table 4 and Supplementary material. All profiling modalities (NMR and in both UPLC-TOF-MS ionisation mode) generated Y-prediction scores with increased predictive accuracy over CPS, CLIF-SOFA, CLIF AD, CLIF-C ACLF and MELD. Combining clinical scores with metabolic profiling methods gave models with similar accuracy to the metabolic profiling techniques and combining CLIF-SOFA (the most accurate clinical scoring system in this cohort) with NMR profiling data gave a high degree of accuracy similar but not superior to NMR profiling (see Table 4).

## Discussion

This study demonstrates that metabolic profiling of plasma in patients with DC can provide both highly accurate prognostication and mechanistic insights into the metabolic and cellular perturbations of acute decompensation. Circulating levels of LPC/PCs, amino acids and energy metabolites combined by multivariate methods in patients who do not survive to hospital discharge have a characteristic profile. For the first time we demonstrate metabotyping to be more accurate than standard clinically derived prognostic tools, although this requires validation in larger datasets.

The most important metabolite class discovered and validated in these prognostic models was the lipid subclass PC/LPC. PC and LPCs have been implicated in malignancies, autoimmune disease, inflammation and cell signalling. These lipids regulate cell senescence, liver repair and lipolysis, and elicit many immune-modulatory functions including enhancing chemotaxis [37], stimulating phagocytosis and upregulating the expression of adhesion molecules [38] and reducing the organ injury and dysfunction in septic shock [39].

Reduced plasma levels of LPCs and PCs occur in murine models of liver injury [40] and human liver failure [41] independently of age, gender and diet and in correlation with MELD [42]. Murine models [40] of alcohol-related liver injury have demonstrated similar differences in the PC/LPC profile, as in our UPLC-TOF-MS results. In particular, the reduction in LPC (16:0/18:0) seen in both our study and in previous reports (in both patients with hepatitis B cirrhosis and alcoholic liver disease) suggest common lipid derangements that correlate with severity of disease and not aetiology.

No previously reported studies provide a mechanistic insight into how PC/LPC are associated with severity of ACLF or outcome. We hypothesised that circulating lipid abnormalities were a reflection of cell death, similar to ALF [25]. Cytokeratin-18 fragments in peripheral blood are generated by apoptosis and full-length cytokeratin-18 generated by necrosis, and are elevated in a variety of liver diseases including NASH and viral hepatitis [43], [44], [45]. The commonly used M30 antibody identifies a fragmented form of cytokeratin-18 generated by cleavage of multiple caspases (3, 6 and 7). The M65 antibody allows measurement of all cytokeratin-18 fragments because of loss of cell membrane integrity from necrosis and/or apoptosis [46]. Concurrent measurement using the M30 and M65 assays allows for the quantification of the relative contributions of apoptosis and necrosis to cell death [46]. The M30/M65 ratio is therefore an indicator of the contribution of apoptosis to the total cell death activity (expressed by M65).

Our hypothesis proved partly correct with strong negative correlations with total cytokeratin-18 and circulating lipids. We demonstrate M30 and M65 levels are increased in patients with DC, compared to stable CLD. In ALF, we have previously demonstrated differences in cross-gut and cross-liver levels of markers of apoptosis [25]. In contrast, and for the first time, we have demonstrated that the liver is a source of total cytokeratin-18 production in DC. This suggests (in conjunction with the strong M65 correlations with lipids (both PC and LDL) and amino acids in the ^1^H NMR spectra) that necrosis is also an important process in ACLF. This is interesting in comparison with recent reports of apoptosis in ACLF [27] and while we agree that apoptosis is an important mechanism of hepatocyte cell death, necrosis may still have an important contribution.

We are not yet in a position to define causality in this relationship, but necrosis may disrupt overall lipid production or its release into peripheral blood, while apoptosis may consume LPC as signalling molecules, as well as contributing to intrahepatic lipid accumulation. LPCs are implicated in the death of a number of endothelial cell types, but in particular of the hepatocyte. In hepatocyte lipoapoptosis in NASH [47], LPC may be produced from di-acylglycerol in preference to triglyceride production if saturated fatty acid (FA) levels are higher than unsaturated FA, or in the presence of pro-apoptotic protein signalling. LPC may then generate apoptosis via mitochondrial-induced caspase activation or activation of G-protein coupled receptors [47]. Exogenous LPC has also been reported to induce apoptosis [47], [48] of endothelial cells and stimulate inflammatory cells [49] in experimental models of sepsis.

Following experimental hepatectomy, hepatocyte loss and acute liver failure is associated with a reduction in levels of circulating lipids and marked hepatic lipid accumulation [50]. A number of recent studies have suggested that hepatic necrosis is also a hallmark of ACLF [51], [52], but as yet there are no human studies that investigate the role of lipids and hepatic necrosis together in ACLF. Nevertheless, formation of TAG within the hepatocyte and failure of LPC production from DAG under the influence of pro-apoptotic proteins may further reduce circulating LPC levels. Following acetaminophen-induced hepatic necrosis in rats, dose dependent increases in serum levels of lactate, pyruvate and isoleucine are noted with reductions in serum GPC [53]. In methotrexate-induced hepatic necrosis in a rat model of NASH using a methionine-choline deficient diet, liver necrosis was associated with higher hepatic GPC [54].

Further studies at the hepatic level with liver tissue are necessary to define a causal relationship between dyslipidaemia and apoptosis/necrosis but both methods of cell death are likely occurring in patients with DC.

We also demonstrated the importance of energy metabolites such as lactate and amino acids such as tyrosine, phenylalanine and methionine. Hepatic ischaemia and failure to clear (as well as increased production of) lactate are well described and lactate is a classic biomarker of mortality in hepatology and critical illness when aerobic metabolism is overwhelmed. Increases in aromatic amino acids are also well described as ammonia levels rise in conjunction with failure of the urea cycle in patients with HE and liver failure [55]. However, ammonia alone is a poor biomarker of survival in cirrhosis as a number of cofactors including inflammation [56] are required to generate high grade HE and contribute to poor outcome. Similarly, the urea rise we see in non-survivors in this study is more likely related to failure of renal excretion given the high creatinine in patients who do not survive. The combination of these markers with lipids in the ^1^H NMR gives similar prognostic accuracy as the UPLC-MS so choosing between the two methods would depend on a number of factors such as cost, reproducibility and familiarity. This is further discussed in the Supplementary material.

The limitations of this study include its relatively small number of participants. We performed sample size analysis, based on previous NMR work in patients with ALF [17], based on a 10% improvement in AUROC curve from an estimated MELD AUROC. Although we used a mix of aetiologies the metabolic profile associated with severe decompensation and mortality has not been shown to be aetiology dependent. Lead-time bias is difficult to fully control in these cohorts, given patients present following decompensation at different times and therefore future validation will include larger patient numbers at different time points during the evolution of decompensation or ACLF.

Deployment of metabolic profiling techniques to assist clinical decision making would require protocols to allow comparison of measurements between centres. While NMR spectrometers would represent a significant capital cost for hospitals each spectral acquisition is possible within 30 min. UPLC-MS instruments are more commonplace, with low space requirements and the ability to quantify metabolite levels in minutes. Translating this technology into the clinical setting is worthwhile, given the potential advantages of both the global profiling techniques and the multivariate statistics [57], [58] to provide highly accurate modelling and a personalised prediction system. This kind of step change in outcome prediction performance can be realised via collaborations, such as the UK National Phenome Centre and Human Phenome Project [59] and may revolutionise approaches to the patient journey based on metabotyping to allow meaningful translation for this powerful but underutilised systems biology toolset.

## Financial support

MJWM was supported by a grant from the Wellcome Trust, London, United Kingdom [090542]. DLS was supported by a HEFCE Clinical Senior Lectureship. EJW acknowledges Waters Corporation for funding. MJWM, MRL, MMEC, EH, and SDTR are grateful to the United Kingdom National Institute for Health Research (NIHR) Biomedical Facility at Imperial College London for infrastructure support and to the Trustees of the Imperial College Healthcare Charity for facilitating sample biobanking. GCA, VP, DS, WB, NH, JOG and JAW acknowledge the support of Medical Research Council (MRC) Centre for Transplantation, King’s College London, UK – MRC grant no. MR/J006742/1 and for support from the National Institute for Health Research (NIHR) Biomedical Research Centre based at Guy’s and St Thomas’ NHS Foundation Trust and King’s College London. The views expressed are those of the author(s) and not necessarily those of the NHS, the NIHR or the Department of Health.

## Conflict of interest

The authors who have taken part in this study declared that they do not have any conflict of interest with respect to this manuscript.

## Authors’ contributions

Designed the study and wrote the protocol MM, DS, STR.

Recruited patients MM, DS, IC, VP, MC, RDA, JOG, NH, WJ, JW, WB, GA.

Performed metabonomics experiments MM, EW, ML, MK, MG.

Analysed metabonomics data MM, EW, ML, KV, MK, EH, MC, RZ.

Performed analysis of cytokeratin 18 data ET, OP, HA.

Performed histological analysis AQ, OP, CGA.

Wrote and/or critically review the manuscript MM, DS, IC, ML, WB, JW, CGA MC, GA, STR, EH, JKN.

Guarantor of the study STR.

## Figures and Tables

**Table 1:** **Demographic, biochemical and physiological details from the derivation study population.**

*p* values – χ^2^ test for categorical variables, Mann Whitney *U* test for continuous variables. Continuous data given as median (range). HE, hepatic encephalopathy; INR, International Normalised Ratio; WCC, white cell count; CPS, Child-Pugh score; MELD, Model for End-Stage Liver Disease; CLIF-SOFA, Chronic Liver Failure Sequential Organ Failure Assessment; UKELD, United Kingdom End-Stage Liver Disease.

**Table 2:** **Nuclear magnetic resonance (NMR) observed metabolites with intensity (mean (SD), arbitrary units) differences associated with decompensated cirrhosis *vs.* controls and those liver disease patients with and without poor prognosis with sign of change denoted.**

The assignment numbers as per the annotation in Fig. 1. Nuclear magnetic resonance peak multiplicity – s (singlet), d (doublet), t (triplet), m (multiplet), dd (doublet of doublets). LDL, low density lipoproteins; *p* values one way ANOVA with multiple comparison correction.

**Table 3:** **The putative identification (ID) of UPLC-TOF-MS measured metabolites associated with cirrhosis and with poor prognosis and intensity differences between classes (mean (SD), arbotrary units).**

LPC, lysophosphatidylcholine; M-H protonated adduct; M-Na sodiated adduct; PC, phosphatidylcholine, choline; PG, phosphatidylglycerol; PI phosphatidylinositol, PS, phosphatyidylserine; metabolite identification superscripted-M-MS/MS, S-standard, A-accurate mass; *p* values one way ANOVA with multiple comparison correction.

**Table 4:** **The comparison of Model for End-Stage Liver Disease (MELD), Child-Pugh Score (CPS) and Chronic Liver Failure Sequential Organ Failure Assessment (CLIF-SOFA), CLIF Acute Decompensation (CLIF AD) and CLIF Acute on Chronic Liver failure (CLIF-C ACLF) for prediction of hospital survival in patients with decompensated cirrhosis in comparison with Y-predicted metabolic profiling strategies via NMR and UPLC-TOF-MS.**

LR, likelihood ratio; MELD, Model for End-Stage Liver Disease; CPS, Child-Pugh Score; CLIF-SOFA, Chronic Liver Failure Sequential Organ Failure Assessment; CPMG, Carr-Purcell-Meiboom-Gill; NMRS, nuclear magnetic resonance spectroscopy; UPLC-MS ESI, Ultra-Performance Liquid Chromatography Mass Spectrometry Electrospray Ionisation; ^Δ^1^st^ validation cohort comparison.

## References

[1] Williams J.G., Roberts S.E., Ali M.F., Cheung W.Y., Cohen D.R., Demery G. (2007). Gastroenterology services in the UK. The burden of disease, and the organisation and delivery of services for gastrointestinal and liver disorders: a review of the evidence. Gut.

[2] Moreau R., Jalan R., Gines P., Pavesi M., Angeli P., Cordoba J. (2013). Acute-on-chronic liver failure is a distinct syndrome that develops in patients with acute decompensation of cirrhosis. Gastroenterology.

[3] Shawcross D.L., Austin M.J., Abeles R.D., McPhail M.J., Yeoman A.D., Taylor N.J. (2012). The impact of organ dysfunction in cirrhosis: survival at a cost?. J Hepatol.

[4] Cholongitas E., Senzolo M., Triantos C., Samonakis D., Patch D., Burroughs A.K. (2005). MELD is not enough–enough of MELD?. J Hepatol.

[5] Cholongitas E., Senzolo M., Patch D., Shaw S., Hui C., Burroughs A.K. (2006). Review article: scoring systems for assessing prognosis in critically ill adult cirrhotics. Aliment Pharmacol Ther.

[6] Jalan R., Saliba F., Pavesi M., Amoros A., Moreau R., Gines P. (2014). Development and validation of a prognostic score to predict mortality in patients with acute-on-chronic liver failure. J Hepatol.

[7] Nicholson J.K., Lindon J.C., Holmes E. (1999). ‘Metabonomics’: understanding the metabolic responses of living systems to pathophysiological stimuli via multivariate statistical analysis of biological NMR spectroscopic data. Xenobiotica.

[8] Trygg J., Holmes E., Lundstedt T. (2007). Chemometrics in metabonomics. J Proteome Res.

[9] Nicholson J.K., Holmes E. (2006). Global systems biology and personalized healthcare solutions. Discov Med.

[10] Clayton T.A., Lindon J.C., Cloarec O., Antti H., Charuel C., Hanton G. (2006). Pharmaco-metabonomic phenotyping and personalized drug treatment. Nature.

[11] Clayton T.A., Baker D., Lindon J.C., Everett J.R., Nicholson J.K. (2009). Pharmacometabonomic identification of a significant host-microbiome metabolic interaction affecting human drug metabolism. Proc Natl Acad Sci USA.

[12] Backshall A., Sharma R., Clarke S.J., Keun H.C. (2011). Pharmacometabonomic profiling as a predictor of toxicity in patients with inoperable colorectal cancer treated with capecitabine. Clin Cancer Res.

[13] Bjerrum J.T., Nielsen O.H., Hao F., Tang H., Nicholson J.K., Wang Y. (2010). Metabonomics in ulcerative colitis: diagnostics, biomarker identification, and insight into the pathophysiology. J Proteome Res.

[14] Williams H.R., Cox I.J., Walker D.G., North B.V., Patel V.M., Marshall S.E. (2009). Characterization of inflammatory bowel disease with urinary metabolic profiling. Am J Gastroenterol.

[15] Shariff M.I., Ladep N.G., Cox I.J., Williams H.R., Okeke E., Malu A. (2010). Characterization of urinary biomarkers of hepatocellular carcinoma using magnetic resonance spectroscopy in a Nigerian population. J Proteome Res.

[16] Ladep N.G., Dona A.C., Lewis M.R., Crossey M.M., Lemoine M., Okeke E. (2014). Discovery and validation of urinary metabotypes for the diagnosis of hepatocellular carcinoma in West Africans. Hepatology.

[17] Saxena V., Gupta A., Nagana Gowda G.A., Saxena R., Yachha S.K., Khetrapal C.L. (2006). ^1^H NMR spectroscopy for the prediction of therapeutic outcome in patients with fulminant hepatic failure. NMR Biomed.

[18] Bales J.R., Bell J.D., Nicholson J.K., Sadler P.J., Timbrell J.A., Hughes R.D. (1988). Metabolic profiling of body fluids by proton NMR: self-poisoning episodes with paracetamol (acetaminophen). Magn Reson Med.

[19] Jimenez B., Montoliu C., MacIntyre D.A., Serra M.A., Wassel A., Jover M. (2010). Serum metabolic signature of minimal hepatic encephalopathy by (1)H-nuclear magnetic resonance. J Proteome Res.

[20] Amathieu R., Nahon P., Triba M., Bouchemal N., Trinchet J.C., Beaugrand M. (2011). Metabolomic approach by 1H NMR spectroscopy of serum for the assessment of chronic liver failure in patients with cirrhosis. J Proteome Res.

[21] Yu K., Sheng G., Sheng J., Chen Y., Xu W., Liu X. (2007). A metabonomic investigation on the biochemical perturbation in liver failure patients caused by hepatitis B virus. J Proteome Res.

[22] Zhang L., Jia X., Peng X., Ou Q., Zhang Z., Qiu C. (2010). Development and validation of a liquid chromatography-mass spectrometry metabonomic platform in human plasma of liver failure caused by hepatitis B virus. Acta Biochim Biophys Sin.

[23] Lian J.S., Liu W., Hao S.R., Guo Y.Z., Huang H.J., Chen D.Y. (2011). A serum metabonomic study on the difference between alcohol- and HBV-induced liver cirrhosis by ultraperformance liquid chromatography coupled to mass spectrometry plus quadrupole time-of-flight mass spectrometry. Chin Med J.

[24] Beyoglu D., Idle J.R. (2013). The metabolomic window into hepatobiliary disease. J Hepatol.

[25] Possamai L.A., McPhail M.J., Quaglia A., Zingarelli V., Abeles R.D., Tidswell R. (2013). Character and temporal evolution of apoptosis in acetaminophen-induced acute liver failure∗. Crit Care Med.

[26] Craig D.G., Lee P., Pryde E.A., Masterton G.S., Hayes P.C., Simpson K.J. (2011). Circulating apoptotic and necrotic cell death markers in patients with acute liver injury. Liver Int.

[27] Adebayo D., Morabito V., Andreola F., Pieri G., Luong T.V., Dhillon A. (2015). Mechanism of cell death in acute-on-chronic liver failure: a clinico-pathologic-biomarker study. Liver Int.

[28] Berry P.A., Wendon J.A. (2006). The management of severe alcoholic liver disease and variceal bleeding in the intensive care unit. Curr Opin Crit Care.

[29] DaWJ Shawcross., Vincent J.L. (2009). Acute-on-chronic liver failure in cirrhosis: defining and managing organ dysfunction. Yearbook of intensive care and emergency medicine 2009.

[30] Pugh R.N., Murray-Lyon I.M., Dawson J.L., Pietroni M.C., Williams R. (1973). Transection of the oesophagus for bleeding oesophageal varices. Br J Surg.

[31] Kamath P.S., Kim W.R. (2007). Advanced Liver Disease Study G. The model for end-stage liver disease (MELD). Hepatology.

[32] Barber K., Madden S., Allen J., Collett D., Neuberger J., Gimson A. (2011). Elective liver transplant list mortality: development of a United Kingdom end-stage liver disease score. Transplantation.

[33] Jalan R., Pavesi M., Saliba F., Amoros A., Fernandez J., Holland-Fischer P. (2015). The CLIF Consortium Acute Decompensation score (CLIF-C ADs) for prognosis of hospitalised cirrhotic patients without acute-on-chronic liver failure. J Hepatol.

[34] Beckonert O., Keun H.C., Ebbels T.M., Bundy J., Holmes E., Lindon J.C. (2007). Metabolic profiling, metabolomic and metabonomic procedures for NMR spectroscopy of urine, plasma, serum and tissue extracts. Nat Protoc.

[35] Smith C.A., Want E.J., O’Maille G., Abagyan R., Siuzdak G. (2006). XCMS: processing mass spectrometry data for metabolite profiling using nonlinear peak alignment, matching, and identification. Anal Chem.

[36] Cummings J., Ranson M., Butt F., Moore D., Dive C. (2007). Qualification of M30 and M65 ELISAs as surrogate biomarkers of cell death: long term antigen stability in cancer patient plasma. Cancer Chemother Pharmacol.

[37] Yun M.R., Okajima F., Im D.S. (2004). The action mode of lysophosphatidylcholine in human monocytes. J Pharmacol Sci.

[38] Kume N., Cybulsky M.I., Gimbrone M.A. (1992). Lysophosphatidylcholine, a component of atherogenic lipoproteins, induces mononuclear leukocyte adhesion molecules in cultured human and rabbit arterial endothelial cells. J Clin Invest.

[39] Murch O., Collin M., Sepodes B., Foster S.J., Mota-Filipe H., Thiemermann C. (2006). Lysophosphatidylcholine reduces the organ injury and dysfunction in rodent models of gram-negative and gram-positive shock. Br J Pharmacol.

[40] Li S., Liu H., Jin Y., Lin S., Cai Z., Jiang Y. (2011). Metabolomics study of alcohol-induced liver injury and hepatocellular carcinoma xenografts in mice. J Chromatogr B Analyt Technol Biomed Life Sci.

[41] Zhang L., Jia X., Peng X., Ou Q., Zhang Z., Qiu C. (2010). Development and validation of a liquid chromatography-mass spectrometry metabonomic platform in human plasma of liver failure caused by hepatitis B virus. Acta Biochim Biophys Sin (Shanghai).

[42] Amathieu R., Nahon P., Triba M., Bouchemal N., Trinchet J.C., Beaugrand M. (2011). Metabolomic approach by 1H NMR spectroscopy of serum for the assessment of chronic liver failure in patients with cirrhosis. J Proteome Res.

[43] Papatheodoridis G.V., Hadziyannis E., Tsochatzis E., Georgiou A., Kafiri G., Tiniakos D.G. (2010). Serum apoptotic caspase activity in chronic hepatitis C and nonalcoholic Fatty liver disease. J Clin Gastroenterol.

[44] Papatheodoridis G.V., Hadziyannis E., Tsochatzis E., Chrysanthos N., Georgiou A., Kafiri G. (2008). Serum apoptotic caspase activity as a marker of severity in HBeAg-negative chronic hepatitis B virus infection. Gut.

[45] Zheng S.J., Liu S., Liu M., McCrae M.A., Li J.F., Han Y.P. (2014). Prognostic value of M30/M65 for outcome of hepatitis B virus-related acute-on-chronic liver failure. World J Gastroenterol.

[46] Kramer G., Erdal H., Mertens H.J., Nap M., Mauermann J., Steiner G. (2004). Differentiation between cell death modes using measurements of different soluble forms of extracellular cytokeratin 18. Cancer Res.

[47] Han M.S., Park S.Y., Shinzawa K., Kim S., Chung K.W., Lee J.H. (2008). Lysophosphatidylcholine as a death effector in the lipoapoptosis of hepatocytes. J Lipid Res.

[48] Takahashi M., Okazaki H., Ogata Y., Takeuchi K., Ikeda U., Shimada K. (2002). Lysophosphatidylcholine induces apoptosis in human endothelial cells through a p38-mitogen-activated protein kinase-dependent mechanism. Atherosclerosis.

[49] Yan J.J., Jung J.S., Lee J.E., Lee J., Huh S.O., Kim H.S. (2004). Therapeutic effects of lysophosphatidylcholine in experimental sepsis. Nat Med.

[50] Bollard M.E., Contel N.R., Ebbels T.M., Smith L., Beckonert O., Cantor G.H. (2010). NMR-based metabolic profiling identifies biomarkers of liver regeneration following partial hepatectomy in the rat. J Proteome Res.

[51] Li H., Xia Q., Zeng B., Li S.T., Liu H., Li Q. (2015). Submassive hepatic necrosis distinguishes HBV-associated acute on chronic liver failure from cirrhotic patients with acute decompensation. J Hepatol.

[52] Cai J., Han T., Nie C., Jia X., Liu Y., Zhu Z. (2016). Biomarkers of oxidation stress, inflammation, necrosis and apoptosis are associated with hepatitis B-related acute-on-chronic liver failure. Clin Res Hepatol Gastroenterol.

[53] Fukuhara K., Ohno A., Ando Y., Yamoto T., Okuda H. (2011). A 1H NMR-based metabolomics approach for mechanistic insight into acetaminophen-induced hepatotoxicity. Drug Metab Pharmacokinet.

[54] Kyriakides M., Hardwick R.N., Jin Z., Goedken M.J., Holmes E., Cherrington N.J. (2014). Systems level metabolic phenotype of methotrexate administration in the context of non-alcoholic steatohepatitis in the rat. Toxicol Sci.

[55] Tessari P., Vettore M., Millioni R., Puricelli L., Orlando R. (2010). Effect of liver cirrhosis on phenylalanine and tyrosine metabolism. Curr Opin Clin Nutr Metab Care.

[56] Shawcross D.L., Sharifi Y., Canavan J.B., Yeoman A.D., Abeles R.D., Taylor N.J. (2011). Infection and systemic inflammation, not ammonia, are associated with Grade 3/4 hepatic encephalopathy, but not mortality in cirrhosis. J Hepatol.

[57] Kinross J.M., Holmes E., Darzi A.W., Nicholson J.K. (2011). Metabolic phenotyping for monitoring surgical patients. Lancet.

[58] Mirnezami R., Kinross J.M., Vorkas P.A., Goldin R., Holmes E., Nicholson J. (2012). Implementation of molecular phenotyping approaches in the personalized surgical patient journey. Ann Surg.

[59] Oetting W.S., Robinson P.N., Greenblatt M.S., Cotton R.G., Beck T., Carey J.C. (2013). Getting ready for the Human Phenome Project: the 2012 forum of the Human Variome Project. Hum Mutat.

