# Supplementary material for: Multivariate metabotyping of plasma predicts survival in patients with decompensated cirrhosis
Source: J Hepatol. 2016 May;64(5):1058–67. doi: 10.1016/j.jhep.2016.01.003 (PMC4876170; doi:10.1016/j.jhep.2016.01.003)
Supplement: Supplementary data [file mmc1.pdf]

Multivariate metabotyping of plasma accurately predicts survival in patients with decompensated cirrhosis

Mark JW McPhail, Debbie L Shawcross, Matthew R Lewis, Iona Coltart, Elizabeth J Want, Charalambos G Antoniades, Kiril Veselkov, Evangelos Triantafyllou, Vishal Patel, Oltin Pop, Maria Gomez-Romero, Michael Kyriakides, Rabiya Zia, Robin D Abeles, Mary ME Crossey, Wayel Jassem, John O'Grady , Nigel Heaton, Georg Auzinger, William Bernal, Alberto Quaglia, Muireann Coen, Jeremy K Nicholson, Julia A Wendon, Elaine Holmes, Simon D Taylor-Robinson

Table of contents

Supplementary methods……………………………………………………………..3

NMR experimental parameters

NMR experimental parameters in the validation experiment

Preprocessing

UPLC-MS experimental parameters

Data processing for UPLC-MS

M30 Immunostaining and histochemistry

Other statistical methods

Supplementary results………………………………………………………………7

Results of comparison of NMR profiling between HC, CLD and DC with permutation analysis

Supplementary Fig. 1

Comparison of 1H NMR profile of patients with and without ACLF

Supplementary Fig. 2

Results of comparison of UPLC-MS analysis of HC, CLD and DC – positive mode ionisation

Supplementary Fig. 3

Confirmation of discriminatory metabolites in negative mode ionisation
Supplementary Fig. 4
Supplementary Fig. 5
Supplementary Fig. 6
Supplementary Fig. 7
Validation cohort table of characteristics. Supplementary Table 1.
Supplementary Fig. 8
Supplementary Fig. 9

Supplementary discussion…………………………………………………………18
Role of cytokeratin-18 breakdown products
LPC and cell death
Application of metabotyping to predicting survival
Supplementary Fig. 10

Supplementary references……………………………………………………….23

Supplementary methods

NMR experimental parameters

A 1D-NOESY presat sequence was performed initially [(RD)-90°-*t*1-90°-*t*m-90°-acquire Free Induction Decay (FID)]. The 90° pulse length was adjusted to approximately 15 µs and 64 transients were acquired into 32K data points. In addition a 1D Carr-Purcell-Meiboom-Gill (CPMG, [RD-90°-(*τ*-180°-*τ*)*n* -acquire FID]) sequence to attenuate the NMR signal of large macromolecules was performed and used for survival analysis. A spin relaxation delay of 64ms was used with water suppression during the relaxation decay. 256 transients were summed over 32K data points. For both sequences, an exponential function was applied to the FID prior to transformation resulting in a Lorentzian line broadening of 0.3 Hz.

NMR experimental parameters in the validation experiment

Plasma samples were thawed, vortexed for a period of 15 seconds and allowed to stand for 10 minutes. The samples were then mixed with phosphate buffer (1:1, 600 µL total volume; 20 % D_2O, 0.142 M $NaHPO_4$, 2 mM NaN_3, 4 mM TSP) and were centrifuged for 15 minutes at 16,000 g at 4°C. The resulting supernatants were then transferred to 5 mm NMR tubes (SampleJet Tube Z105684; Bruker) for NMR spectral acquisition. A standard one-dimensional solvent suppression pulse sequence and CPMG spin-echo pulse sequence were used to acquire the FID.

For the CPMG 128 scans were performed into 74K data points using a 4s relaxation delay 3.06 s acquisition time and 0.3line broadening.

For the second validation cohort CPMG 128 scans were performed into 32K data points using a 4s relaxation delay 3.06 s acquisition time and 0.3line broadening.

Preprocessing

All spectra were phased and baseline corrected within TopSpin (version 2.1, Bruker Biospin GmbH, Rheinstetten, Germany) and the chemical shifts referenced to the α-glucose anomeric resonance at δ 5.23. Data were transferred into MATLAB (version

7, The Mathworks, Inc.; Natwick, MA, USA) for alignment (using recursive wave peak method) and normalised (using probabilistic quotient method) using routines developed in-house (K. Veselkov). The region between δ 4.5 and δ 5.1 containing the water peak was removed. The resulting full resolution data matrices were further analyzed using SIMCA-P (version 12.0.1 UMETRICS AB, Umeå, Sweden). Pareto-scaling and mean centering were performed prior to multivariate analysis.

UPLC-MS experimental parameters

Starting with an eluent composed of 99.9% water (Ultra Purity Solvent Water, Romil Ltd, Cambridge, UK) plus 0.1% formic acid (Sigma-Aldrich, Gillingham, UK), and 0.1% methanol (Ultra Purity Solvent Methanol, Romil Ltd, Cambridge, UK) plus 0.1% formic acid, gradually reversing over 26 min prior to a 4 min return to original conditions. The temperature was maintained at 50°C on a Waters Acquity UPLC HSS T3 column (1.8 µm, 2.1 × 100 mm) during chromatography. Tandem time of flight (TOF) mass spectrometry (MS) was performed using an electrospray injection (ESI) ionisation operating in both positive and negative modes. ESI conditions were source temperature 120°C, desolvation temperature 400°C, cone gas flow 25L/h, desolvation gas 800L/h, capillary voltage for ESI- 2400V, for ESI +ve 3000 V, cone voltage 25 V. Each injection was of 3µL. At the start of acquisition ten conditioning QC injections were performed and after every 10th subsequent injection. Data were collected in centroid mode. Regular injections of leucine enkephalin (555.2692 Da calculated monoisotopic molecular weight, 200pg/uL in acetonitrile:water 50:50) were performed to ensure optimum mass accuracy with an analyte-to-reference scan ratio of 10:1. Instrument calibration was with sodium formate (10ng/uL in 90:10 propan-2-ol:water) solution prior to each ESI mode.

Identification strategies for NMR and UPLC-MS data

In NMR, corroborative experiments on an 800MHz Bruker spectrometer using 1D NOESY and CPMG sequences were performed together with 2D ^{1}H-^{1}H J-resolved and 2D ^{1}H-^{13}C Heteronuclear Single-Quantum Correlation (HSQC) experiments. Statistical Total Correlation Spectroscopy (STOCSY)) [1]was used to explore high multi-co linearity amongst spectral intensities for both metabolite identification and exploration of potential biochemical pathways involved in class discrimination. A QC

sample from the UPLC-MS well plate was also analysed under identical UPLC gradient conditions in a quadruple time-of-flight (QTOF) MS-MS instrument (Waters Corporation, Milford, MA, USA) to aid confident identification of metabolites from the fragmentation pattern, in conjunction with the use of online databases and standards. . Lastly, purified standards of selected lipids were obtained from Avanti (Alabaster, Alabama) and analysed under identical UPLC-MS conditions to further aid identification.

Data processing for UPLC-MS

Data were processed prior to multivariate analysis using the XCMS software suite in the R programming language (http://www.bioconductor.org/biocLite.R). Following conversion to CDF format, MS data underwent peak picking, grouping, and retention time correction. Peaks identified (by retention time and mass) were compared back to the original data during processing. Samples were grouped by clinical class with QC samples treated as a separate group.

M30 Immunostaining and histochemistry

FFPE tissue was cut at 4 μm using a Leica RM2235 rotary microtome (Leica Biosystems, UK) and picked up on poly-l-lysine coated slides which were dewaxed in xylene, rehydrated, subjected to heat-induced epitope retrieval (HIER) using sodium citrate buffer, pH 6, for 20 minutes, and allowed to cool down to room temperature. Endogenous avidin and biotin blocking steps were performed for 15 minutes each using an avidin/biotin blocking kit (catalog number SP-2001, Vector Laboratories, UK), followed by the incubation with the primary antibody for 2 hours at room temperature, a 40 minutes incubation with the biotinilated secondary antibody (product number RE7103, Leica Biosystems, UK), and another 40 minutes incubation with horseradish peroxidase-conjugated streptavidin (product number RE7104, Leica Biosystems, UK). The Peroxidase Substrate Kit (Vector® VIP) was used to visualize the signal (catalog number SK-4600, Vector Laboratories, UK). All slides were counter-stained with Carazzi's haematoxylin for 10 seconds, then dehydrated with alcohol, cleared with xylene and mounted with DPX (Leica Biosystems, UK).

Slides were imaged using an Olympus BX53 microscope equipped with an Olympus DP26 digital camera, using the cellSens Entry 1.9 software (Olympus, UK).

Immunostains were analyzed by a liver histopathologist (A. Q.) who was blinded to the clinical data. Cell counts of M30+ cells as well as acidophilic bodies on H & E stains were carried out using the ImageJ software (Bethesda, Maryland, USA) on 10 high power fields (HPF) from hepatic plates.

No present M65 immunostaining methods are available for histochemistry for total cytokeratin-18.

Other statistical methods

Continuous data were expressed as mean(standard deviation) or median (range) contingent on results of normality testing by the Agostino-Pearson method. Student's t-test/one way ANOVA or MannWhitney U test/Kruskall Wallis test was then performed as appropriate. Categorical data were analysed by $\chi 2$ test. Data were analysed in MedCalc (MedCalc Software, Mariakerke, Belgium) and GraphPad Prism v 6 (Graphpad software, La Jolla CA USA) in addition to MATLAB v8 (Mathworks, Natick, MA, USA), R and SIMCA v13 (Umetrics, Umea, Sweden).

Supplementary results

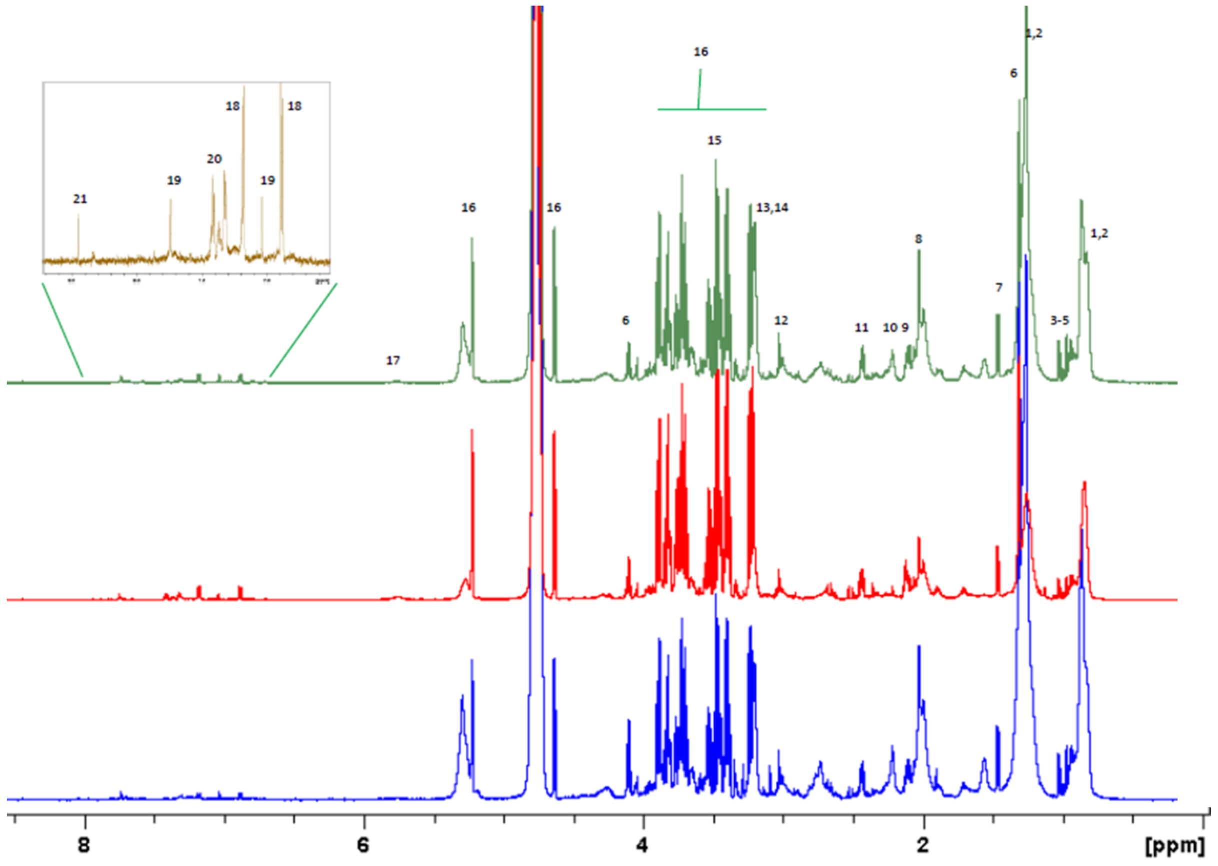

Supplementary Fig. 1: ^{1}H 600 MHz water suppressed CPMG (Carr Purcell Meiboom Gill) nuclear magnetic resonance plasma spectra from the investigated cohort (A) Healthy control (B) Non-surviving patient with cirrhosis (DC-D) (C) Surviving patient with cirrhosis (DC-S) Metabolites 1-20 described in the main article as per the annotation in the first column of Table 2.

Results of comparison of NMR profiling between HC, CLD and DC with permutation analysis

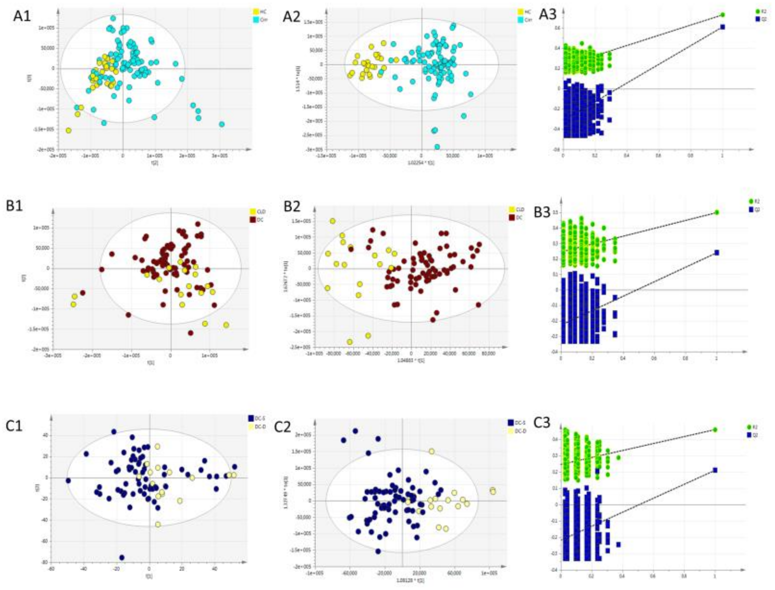

Supplementary Fig. 2: *Results of 1H NMR profiling of plasma. Principal components analysis (PCA) and orthogonal projection least squares discriminant analysis (OPLSDA) Scores plots for the following comparisons: 1) Healthy controls (HC) v patients with cirrhosis (Cirr) A1)PCA $R^2X=0.75$ $Q^2=0.54$ A2) OPLSDA $R^2X=0.67$ $R^2Y=0.75$ $Q^2=0.59$ A3) associated permutation test 2) Patients compensated chronic liver disease (CLD) versus patients with decompensated cirrhosis (DC) (B1) PCA $R^2X=0.62$ $Q^2=0.56$ (B2) OPSLDA $R^2X=0.56$ $R^2Y=0.50$ $Q^2=0.19$, (B3) associated permutation test and 3) Patients with decompensated cirrhosis who died (DC-D) or survived (DC-S) (C1) PCA $R^2X=0.75$ $Q^2=0.54$ (C2) OPSLDA $R^2X=0.57$ $R^2Y=0.46$ $Q^2=0.25$, (C3) associated permutation test.*

Comparison of 1H NMR profile of patients with and without ACLF

In the patients with cirrhosis it was possible to make a valid model discriminating patients with and without ACLF. An OPLSDA model had the following statistics ($R^2X=0.25$, $R^2Y=0.49$, $Q^2Y=0.38$, CV-ANOVA 10^{-9}, sensitivity 83%, specificity 90%). Permutation testing demonstrated that the model was valid in that cutoffs for R2 and Q2 were lower in the randomly permutated model (999 permutations).

Discriminant metabolites were GPC, LDL and VLDL which were lower in the ACLF group and lactate and valine elevated in patients with ACLF.

These data are shown in Supplementary Fig. 3.

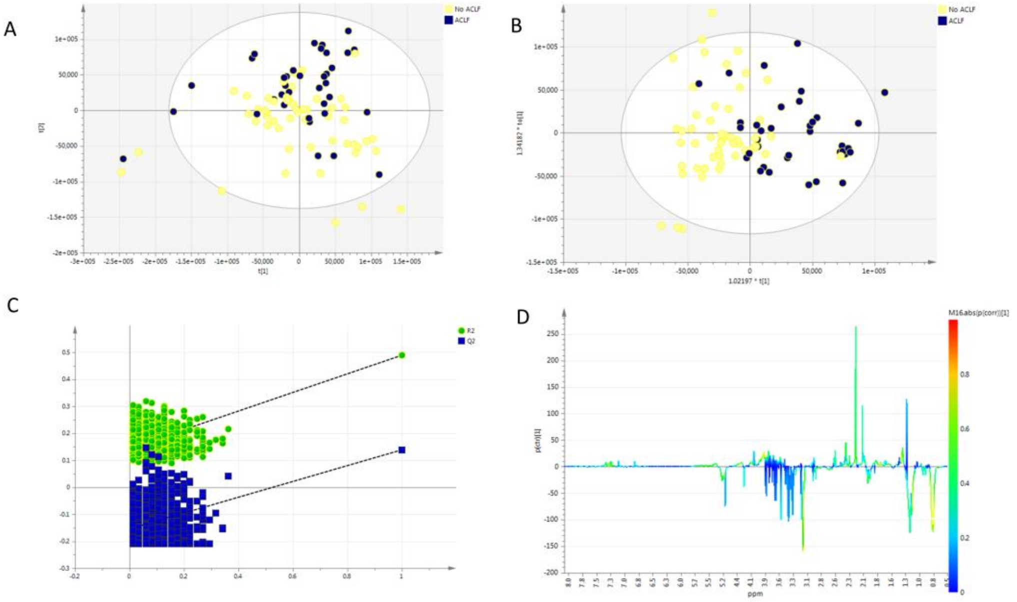

Supplementary Fig. 3 A) PCA plot demonstrating clustering by presence or absence of ACLF in the plasma 1H NMR spectra. B) OPLSDA (1+1+0) demonstrating clear discrimination between ACLF and no ACLF patents 1H NMR spectra. C) Permutation testing for a PLSDA model with the same components as B). D) S loading plot demonstrating metabolite perturbation in patients with and without ACLF.

It was not possible to discriminate patients with varying grades of ACLF due to small numbers in the individual subgroups.

Results of comparison of UPLC-MS analysis of HC, CLD and DC – positive mode ionisation

The results of comparison by PCA and OPLSDA for the dataset from positive mode ionisation are shown in Supplementary Fig. 3 as described in the main document.

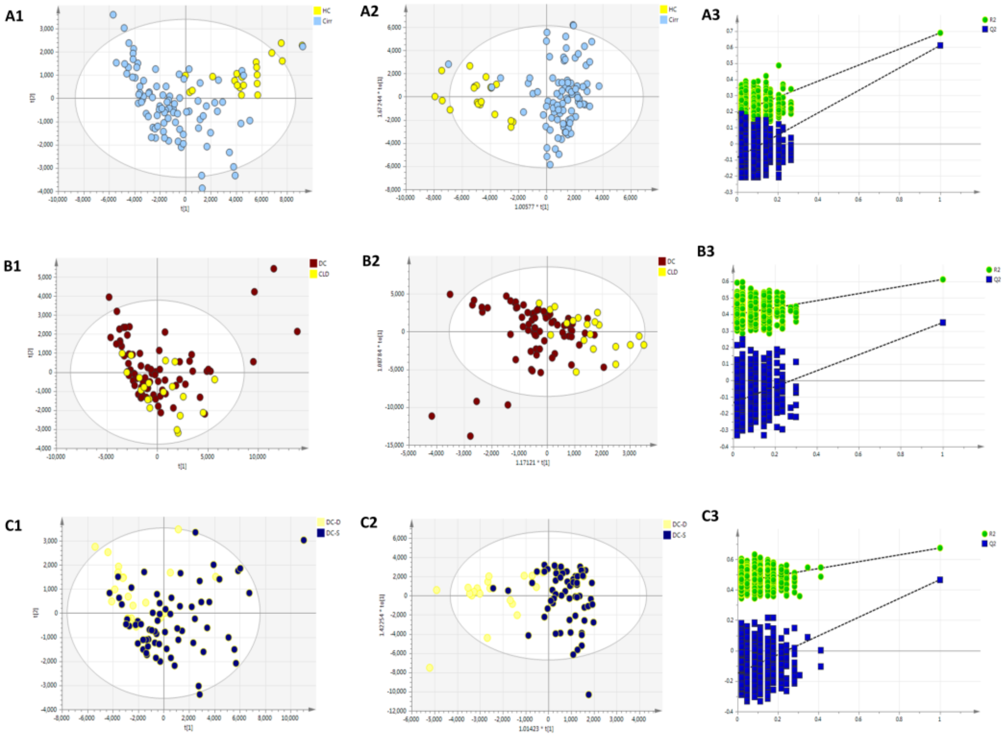

Supplementary Fig. 4: Ultra performance liquid chromatography mass spectrometry positive mode ionisation multivariate analysis: Principal components analysis (PCA) and orthogonal projection least squares discriminant analysis (OPLSDA) Scores plots for the following comparisons: A) Healthy controls (HC) v patients with cirrhosis (Cirr) A1)PCA R^2X=0.53 Q^2=0.51 A2) OPLSDA R^2X=0.52 R^2Y=0.69 Q^2=0.60, A3) Permutation analysis B) Patients compensated chronic liver disease (CLD) versus patients with decompensated cirrhosis (DC) B1) PCA R^2X=0.57 Q^2=0.47 B2) OPSLDA R^2X=0.53 R^2Y=0.61 Q^2=0.26) B3 Permutation analysis C) Patients with decompensated cirrhosis who died (DC-D) or survived (DC-S) C1) PCA R^2X=0.54 Q^2=0.42 C2) OPSLDA R^2X=0.52 R^2Y=0.67 Q^2=0.42 C3) Permutation analysis

Confirmation of discriminatory metabolites in negative mode ionisation

In the dataset produced by negative ion formation (examples in Fig. 3), a three component PCA model only described 47% of the variation in X (R^2X =0.47) with a Q^2Y of 0.42. In terms of modelling ability with supervised techniques, ESI-ve data gave

a similar performance to the dataset from positive ion information from OPLS-DA models, comparing healthy controls and cirrhosis patients (R^2X=0.42, R^2Y=0.69, Q^2Y=0.55, AUROC 0.97, sensitivity 100%, specificity 97%, CV-ANOVA p=10^{-15}) and between survivors and non-survivors (R^2X=0.30, R^2Y=0.58, Q^2Y=0.41, AUROC 0.94, sensitivity 90%, specificity 100%, CV-ANOVA p=10^{-8}). Permutation testing further confirmed the validity of these models. Details on putative metabolite identification are given in Table 3 in the main article. Several discriminant phosphocholine moieties detected in the positive ion dataset were confirmed in negative mode data. Omparison of from accurate mass and fragmentation pattern of all published mass databases and remains unknown at this stage. A mass consistent with the glucuronide conjugate of the anaesthetic medication, propofol, was identified by accurate mass and MS/MS. Adducts related to this molecule were removed from all reported models.

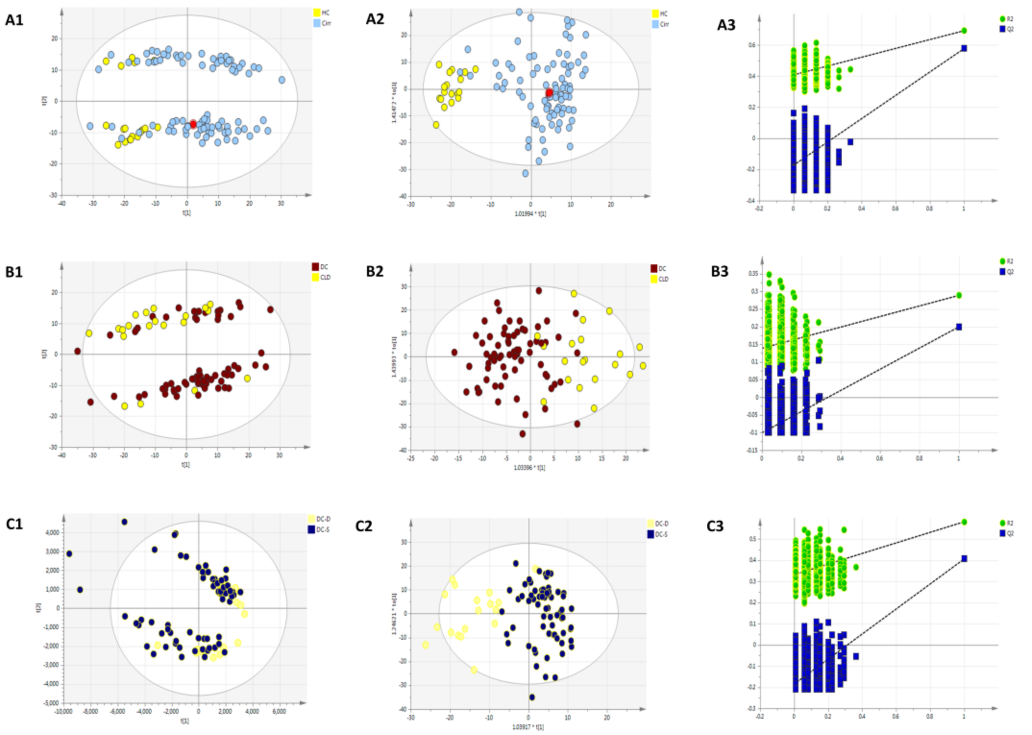

Supplementary Fig. 5: *Ultra performance liquid chromatography mass spectrometry negative mode ionisation multivariate analysis: Principal components analysis (PCA) and orthogonal projection least squares discriminant analysis (OPLSDA) Scores plots for the following comparisons: A) Healthy controls (HC) v patients with cirrhosis (Cirr) A1)PCA $R^2X=0.47$ $Q^2=0.43$ A2) OPLSDA $R^2X=0.42$ $R^2Y=0.69$ $Q^2=0.55$, A3) Permutation analysis B) Patients compensated chronic liver disease (CLD) versus patients with decompensated cirrhosis (DC) B1) PCA $R^2X=0.45$ $Q^2=0.39$ B2) OPSLDA $R^2X=0.39$ $R^2Y=0.55$ $Q^2=0.19$), B3) Permutation analysis C) Patients with decompensated cirrhosis who died (DC-D) or survived (DC-S) C1) PCA $R^2X=0.48$ $Q^2=0.38$ C2) OPSLDA $R^2X=0.37$ $R^2Y=0.71$ $Q^2=0.34$, C3) Permutation analysis. The PCA plots show evidence of batch effect due to a change in chromatographic conditions during the run.*

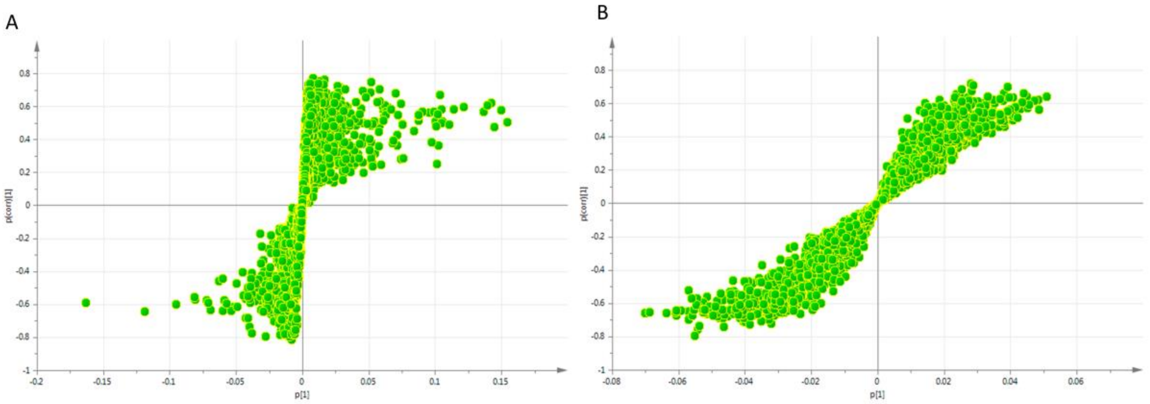

Supplementary Fig. 6. S loading plots for OPLSDA models for discrimination of patients with DC who survived or did not survive in A) positive mode ionisation and B) negative mode ionisation.

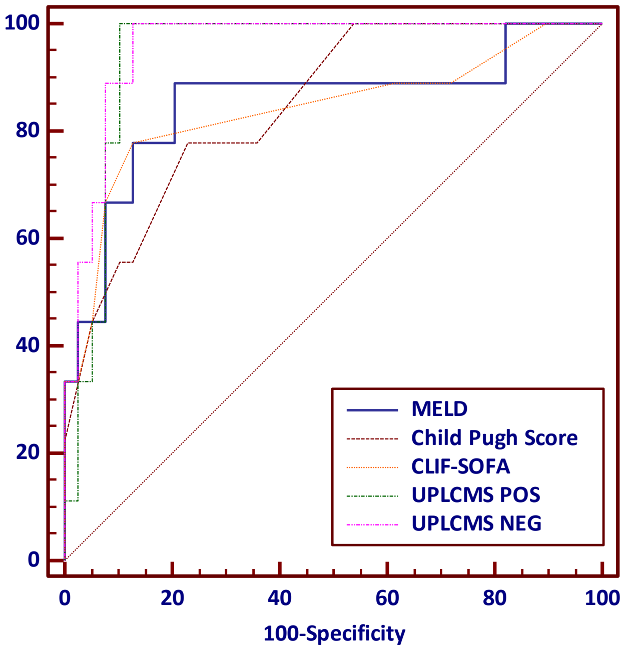

Supplementary Fig. 7: B)UPLCMS positive mode ions profile (ESI +ve Y PREDICTED - AUROC 0.94(0.89-0.98), sensitivity 100%, specificity 85%, p<0.001); UPLCMS negative mode ions profile (ESI -ve Y PREDICTED - AUROC 0.94(0.88-0.98) sensitivity 100%, specificity 85%, p<0.001); CLIF-SOFA - AUROC 0.87(0.77-0.93) sensitivity 78%, specificity 91%, p<0.001); MELD - AUROC 0.81(0.66-0.96), sensitivity 78%, specificity 86%, p<0.001); Child-Pugh Score - AUROC 0.87 (0.76-0.98), sensitivity 83%, specificity 78%, p<0.001.

Validation cohort results$^{\Delta}$

Supplementary Table 1: Demographic, biochemical and physiological details from the validation study population ∆ primary validation cohort. p-values - χ2 test for categorical variables, Mann Whitney U test for continuous variables. Continuous data given as median (range). Abbreviations HE (Hepatic Encephalopathy), INR (International Normalised Ratio), WCC (White Cell Count), CPS (Child Pugh Score), MELD (Model for End Stage Liver Disease), CLIF-SOFA (Chronic Liver Failure Sequential Organ Failure Assessment) UKELD (United Kingdom End Stage Liver Disease)

Variable	All patients	Survivors	Non-survivors	p-value
	59	40	19	
**Age**	50(13)	49(13)	52(8.6)	0.354
**Sex (Male:Female)**	39:20	25:15	14:5	0.579
**Aetiology**				
**Alcohol**	31	23	8	0.463
**Viral hepatitis**	0	0	0	
**Autoimmune**	9	5	4	
**Non-alcoholic steatohepatitis**	11	6	5	
**Other**	8	7	1	
**Serum Na (mmol/L)**	137(5)	136(5)	137(6)	0.346
**K (mmol/L)**	4.3(0.6)	4.2(0.6)	4.5(0.7)	0.103
**Ct (µmol/L)**	115(20-693)	88(20-693)	162(82-331)	<0.001
**AST (iU/L)**	88(23-8896)	59(23-1670)	137(28-8896)	0.065
**GGT (U/L)**	63(13-2423)	79(18-2423)	47(13-1114)	0.412
**Bilirubin (µmol/L)**	98(12-603)	70(10-714)	209(20-603)	0.001
**Albumin (g/L)**	28(7)	30(6)	24(8)	0.014
**INR**	1.7(1.1-7.7)	1.5(1.1-3.7)	2.1(1.3-7.7)	<0.001
**Haemoglobin (g/L)**	10.1(1.9)	10.4(2.1)	9.5(1.5)	0.113
**WCC (x 10^9/L)**	7.7(1.6-26.8)	8.1(1.6-26.8)	11.2(1.6-25)	0.008
**Platelets (x 10^9/L)**	100(19-479)	102(33-479)	81(19-229)	0.178
**HE Grade**	2(0-4)	1(0-4)	3(1-4)	<0.001

**Ammonia (µmol/L)**	66(18-300)	67(18-300)	64(5-133))	0.567
**Mechanically ventilated(Y:N)**	21:38	6:34	15:4	<0.001
**Vasopressor use(Y:N)**	23:36	8:32	15:4	<0.001
**CPS**	11(6-14)	10(6-13)	13(10-14)	<0.001
**CLIF-SOFA**	12(3-22)	8(3-23)	17(12-25)	<0.001
**MELD**	23(6-40)	13(6-40)	37(11-40)	<0.001
**UKELD**	58(6)	56(6)	61(5)	0.002

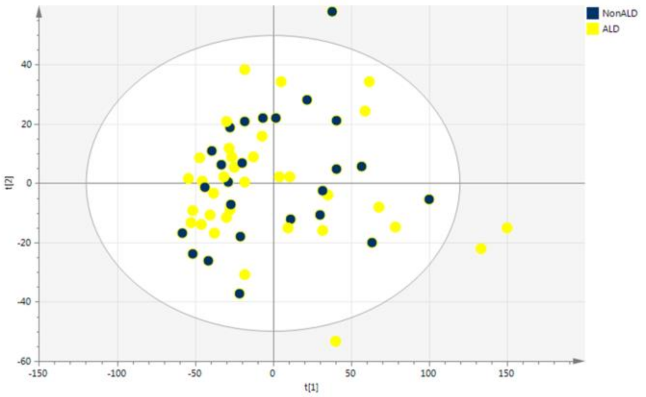

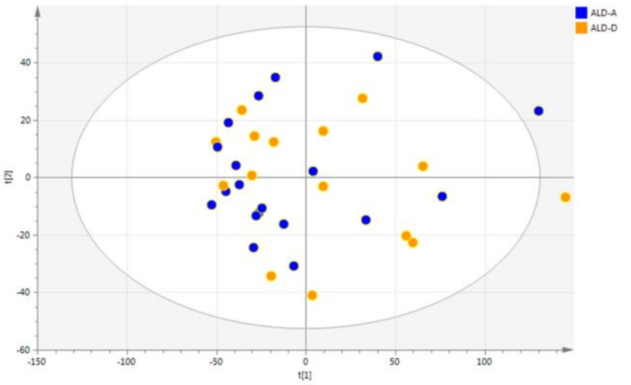

Supplementary Fig. 8: Principal component analysis (PCA) comparing 1H NMR spectra of plasma in patients with A) (ALD) or without (nonALD) alcohol as the underlying aetiology of cirrhosis in the 1st validation cohort (R2X=0.61, Q2Y=0.55) and B) comparing abstinent (ALD-A) versus non abstinent (ALD-D) patients,

R2X=0.65 Q2Y=0.53). The visual impression is these groups are not discriminated and this is borne out in supervised modelling where no valid models could be built.

Second validation cohort

A second metabonomics validation analysis was performed using previously stored samples. These were taken, stored and analysed by 1H NMR as per the derivation cohort. They were comprised of 27 healthy controls and 42 patients with cirrhosis. The patients with cirrhosis had a median age of 43 (27-86) years and 27(64%) were men. The aetiology of cirrhosis was alcohol in 25, viral hepatitis in 4, NASH (metabolic) in 6 and unknown in the remainder. 14 died within the 90 day follow up period and patients undergoing transplantation were excluded. The median MELD score was 19(6-40). The metabonomics analysis is presented in Supplementary Fig. 9.

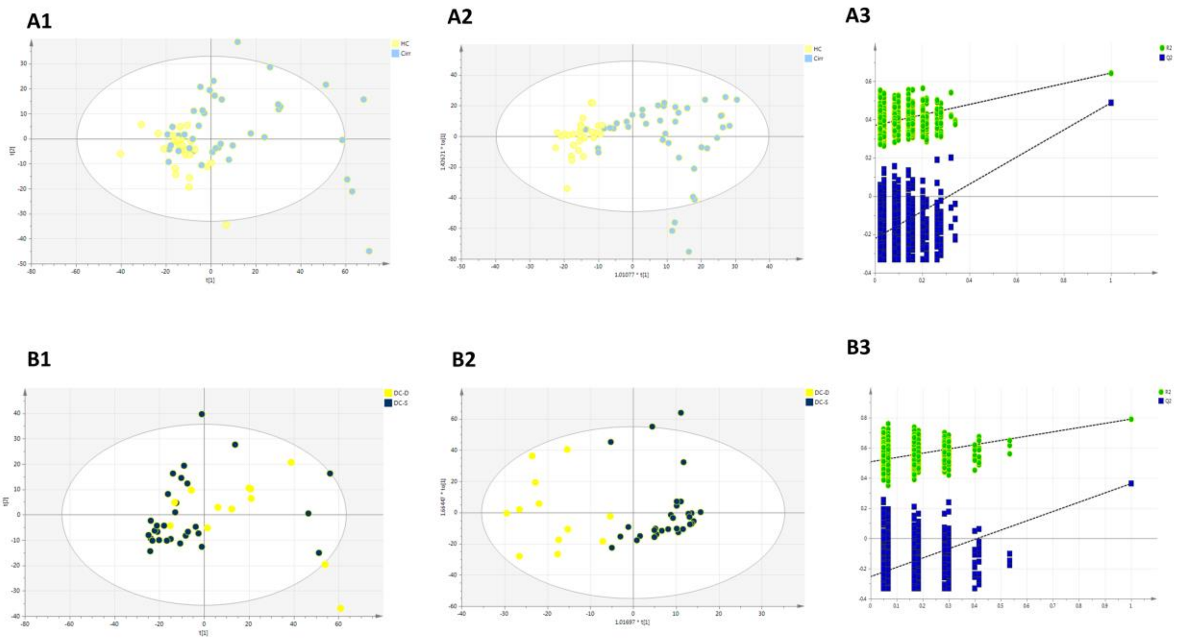

Supplementary Fig. 9: 1H NMR multivariate analysis of second validation cohort: Principal components analysis (PCA) and orthogonal projection least squares discriminant analysis (OPLSDA) Scores plots for the following comparisons: A) Healthy controls (HC) v patients with cirrhosis (Cirr) A1) PCA R^2X=0.61 Q^2=0.50 A2) OPLSDA R^2X=0.56 R^2Y=0.64 Q^2=0.56, A3) Permutation analysis B) Patients with decompensated cirrhosis who died (DC-D) or survived (DC-S) B1) PCA R^2X=0.61 Q^2=0.47 B2) OPSLDA R^2X=0.51 R^2Y=0.79 Q^2=0.48, B3) Permutation analysis.

The ability of the CPMG 1H NMR profile to predict mortality was high with an AUROC of 0.95(0.88-0.99, sensitivity 100(75-100)%, specificity 91(75-98)%, LR+ 11(3.7-32), LR- 0) in comparison with MELD, AUROC 0.85(0.74-0.93), sensitivity 100(77-67)%, specificity 67(47-82), LR+ 3.0(1.8-5), LR- 0)) and CLIF C AD (AURIC 0.90(0.79-1.00), sensitivity 69(38-91), 100(79-100), LR- 0.31 (0.1-.0.7))

Supplementary Discussion

Role of cytokeratin-18 breakdown products

Plasma concentrations levels of cytokeratin-18 act as a circulating biomarker of the mechanism of cell death. Cytokeratin-18 is a cytoskeletal intermediate filament protein found in simple epithelial cells, and together with keratin-8 are the only keratin intermediate filaments in hepatocytes. Cytokeratin-18 fragments in peripheral blood are generated by apoptosis and full-length cytokeratin-18 generated by necrosis, and while detectable in serum of non-liver disease controls[2], are elevated in a variety of liver diseases including NASH and viral hepatitis [3-5]. The commonly used M30 antibody identifies a fragmented form of cytokeratin-18 which is an apoptosis-specific neo-epitope at the cytokeratin-18 aspartic acid residue 396, generated by caspase-6, caspase-3 and caspase-7 cleavage. The M65 antibody allows for measurement of all cytokeratin-18 fragments because of loss of cell membrane integrity from necrosis and/or apoptosis[6]. Thus, concurrent measurement using the M30 and M65 assays allows for the quantification of the relative contributions of apoptosis and necrosis to cell death [6]. The M30/M65 ratio is therefore an indicator of the contribution of apoptosis to the total cell death activity. M65 levels do not reflect pure necrosis but cell death as a whole.

Our results suggest that both apoptosis and necrosis are occurring at the hepatocyte level in patients with DC. The closer correlation with M65 and lipids measured by metabolic profiling suggests that the necrosis element of cell death has more in common mechanistically with the metabolic profiling data and to the clinical outcome. Nevertheless, there is evidence for ongoing hepatocyte apoptosis both from the M30

levels and apoptotic specific staining therefore both processes contribute to critical loss of hepatic function in DC.

LPC and cell death

LPCs are implicated in the death of a number of endothelial cell types but in particular of the hepatocyte in NASH. In lipoaptosis[7] LPC may be produced from diacylglycerol in preference to trglyceride production if saturated fatty acid concentrations are higher than unsaturated fatty acids. LPC may then generate apoptosis via mitochondrial induced caspase activation or activation of G-protein coupled receptors.

Exogenous LPC has been reported to induce apoptosis [7, 8] of endothelial cells and stimulate inflammatory cells [9] in experimental models of sepsis. Plasma concentrations of other phospholipids, such as phosphatidylethanolamine, phosphatidylglycerol, phosphatidylinositol, and phosphatidylserine, are less than one-tenth of that of PC[10]. Much higher in vivo concentrations and much stronger apoptotic activity of LPC compared with LPE, LPG, LPI, or LPS reduces the possibility that other lysophospholipids play significant roles in lipoapoptosis. While previous studies report on the apoptotic activity of LPC, that of other lysophospholipids has not been clearly demonstrated [8]. Following apoptotic signaling diacylglycerols (DAG)s may preferentially form triacylglycerols (TAG) within the cell cytoplasm and do not participate in further LPC production further reducing the levels of LPCs.

We are not suggesting that cell death mechanisms alone are implicated in the reduction of serum LPC we see here. ACLF is also associated with high levels of pro-inflammatory cytokines and failure of CARS response in patients who do not survive. Levels of short chain fatty acid LPC (eg 16:0 and 18:0) have been shown to decrease under pro-inflammatory conditions[11] when mesenchymal stromal cells are exposed to TNF-a and IFN-g. LPC pre-treatment in animal models of sepsis reduced pro-inflammatory cytokine production, positive blood culture rates and mouse mortality[12]. Furthermore in human ACLF circulating gDNA act as a DAMP inducing pro-inflammatory genes and promoting further apoptosis[13].

Finally enhanced faecal excretion of major LPC have been demonstrated in patients with cirrhosis [14] enhancing the possible role of gut microbes in this core phenotypic change seen in chronic liver disease.

Therefore, inflammation and the role of gut host-microbe interactions are further important co-factors in these patients which requires further study.

Application of metabotyping to predicting survival

The general approach we recommend would be to determine a metabotype of mortality from a large body of samples with known outcomes. Then the position of the profile of an individual patient on the principal component or latent variable can be assessed to determine the probability of which class (alive or died) the new patient would lie. The profile used could be that of the entire spectrum or of the combinations of a smaller set of metabolites as we propose. The former has the benefit of including all metabolites including those which may only be of interest in the minority of patients whereas the limited profile approach has the benefit of being easier to understand and only requires the concentrations of the metabolites of interest. The former may be more suited to an NMR based application and the latter to a UPLCMS methodology. We would recommend using an OPLSDA model to determine the “position” of the patient of interest on the metabotype cross validated Y prediction score against the bank of previous samples.

The potential weakness in this approach could be the different measurement platforms utilised by different medical centres for these metabolites as well as differing medical management affecting the profile itself. We use Bruker NMR spectrometers and Waters mass spectrometers and therefore reapplication of our techniques on these commonly used instruments would be straightforward. Were a limited set of metabolites validated for use based on absolute quantification it may be that these measures could become platform independent as long as agreed quality assurance methods were undertaken.

Furthermore, when this list of metabolites is known or further refined standard logistic regression modelling could be performed to assess if these are comparable to PCA/OPLSDA methods. In such a scheme the multivariate methods we use here become more useful for the discovery of biomarkers of interest rather than the predictive tools themselves. Applications such as the “Intelligent Knife” at Imperial

College use both spectroscopic data and PCA/OPLSDA analysis for prediction in real time so this is already being used in clinical studies.[15]

Clinicians may be less conversant with these multivariate methods compared to logistic regression and require PCA/PLSDA to be performed in the background with the summary result presented. It is possible to perform PCA on handheld apps and so the opportunity to develop a web or mobile based metabotyping app exists. For example, the XCMS mobile app allows a large part of the functionality of PCA of mass spectrometry data to be performed on a mobile device. Similar front end technology would need to be developed to allow profiling data or metabolite concentrations to be entered and a risk of death calculated. We are developing such technology and hope to assess it using both spectroscopic and non-spectroscopic data in the future.

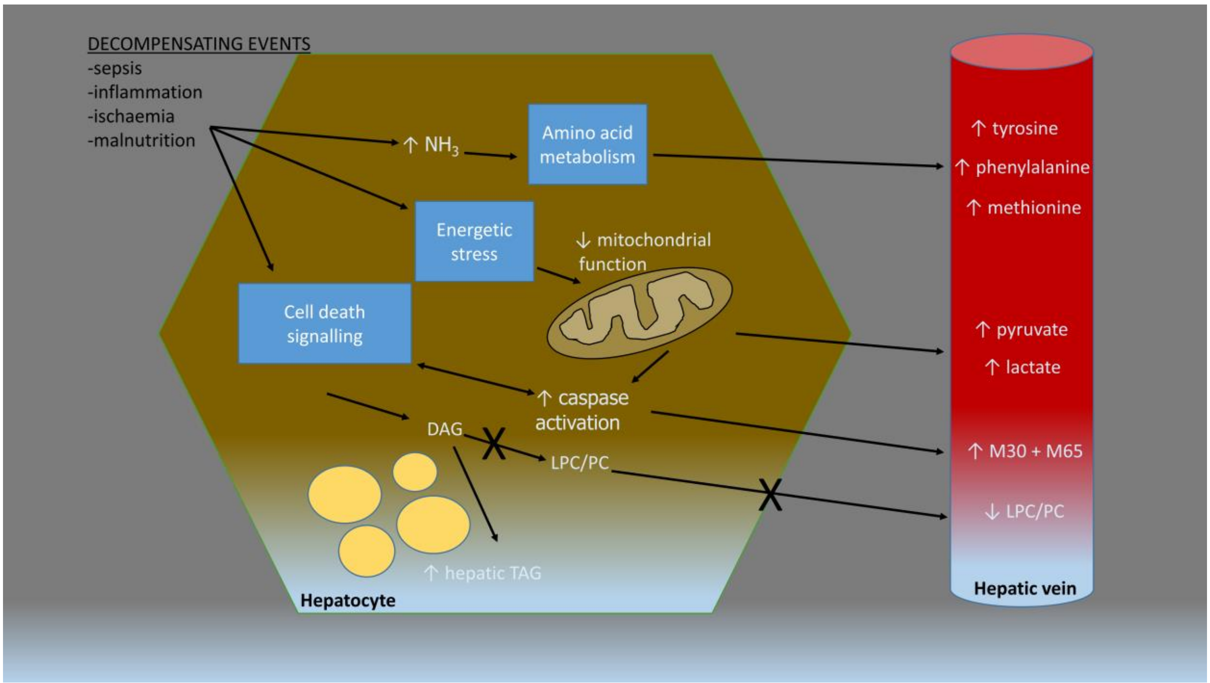

Supplementary Fig. 10: Postulated mechanisms for metabolite derangement in decompensated cirrhosis. Following the precipitation for decompensation three main mechanisms are invoked. Hyperammonaemia causes partial failure of the urea cycle and preferential production of aromatic amino acids causes higher peripheral blood levels while branch chain amino acid levels fall. Hepatic ischaemia and glycolysis causes both overproduction of lactate and failure of clearance and peripheral blood lactate and pyruvate levels rise. The hepatocyte undergoes apoptosis/necrosis

resulting in induction of caspases and cleavage of cytokeratin-18. Free fatty acids (FFA) are preferentially converted into diacylglycerols (DAG) which in the presence of cell death signalling preferentially form TAG (triacylglycerides) which accumulate in the hepatocyte cytoplasm and do not form LPC/PC. Intrahepatic lipid accumulates but do not pass into peripheral blood possibly under the influence of low ATP levels. Peripheral levels of LPC and PC therefore reduce when hepatocytes undergo the above stressors and apoptosis/necrosis.

Supplementary references

[1] Cloarec O, Dumas ME, Craig A, Barton RH, Trygg J, Hudson J, et al. Statistical total correlation spectroscopy: an exploratory approach for latent biomarker identification from metabolic 1H NMR data sets. Anal Chem 2005;77:1282-1289.

[2] Omary MB, Ku NO, Strnad P, Hanada S. Toward unraveling the complexity of simple epithelial keratins in human disease. J Clin Invest 2009;119:1794-1805.

[3] Papatheodoridis GV, Hadziyannis E, Tsochatzis E, Georgiou A, Kafiri G, Tiniakos DG, et al. Serum apoptotic caspase activity in chronic hepatitis C and nonalcoholic Fatty liver disease. J Clin Gastroenterol 2010;44:e87-95.

[4] Papatheodoridis GV, Hadziyannis E, Tsochatzis E, Chrysanthos N, Georgiou A, Kafiri G, et al. Serum apoptotic caspase activity as a marker of severity in HBeAg-negative chronic hepatitis B virus infection. Gut 2008;57:500-506.

[5] Zheng SJ, Liu S, Liu M, McCrae MA, Li JF, Han YP, et al. Prognostic value of M30/M65 for outcome of hepatitis B virus-related acute-on-chronic liver failure. World J Gastroenterol 2014;20:2403-2411.

[6] Kramer G, Erdal H, Mertens HJ, Nap M, Mauermann J, Steiner G, et al. Differentiation between cell death modes using measurements of different soluble forms of extracellular cytokeratin 18. Cancer Res 2004;64:1751-1756.

[7] Han MS, Park SY, Shinzawa K, Kim S, Chung KW, Lee JH, et al. Lysophosphatidylcholine as a death effector in the lipoapoptosis of hepatocytes. J Lipid Res 2008;49:84-97.

[8] Takahashi M, Okazaki H, Ogata Y, Takeuchi K, Ikeda U, Shimada K. Lysophosphatidylcholine induces apoptosis in human endothelial cells through a p38-mitogen-activated protein kinase-dependent mechanism. Atherosclerosis 2002;161:387-394.

[9] Yan JJ, Jung JS, Lee JE, Lee J, Huh SO, Kim HS, et al. Therapeutic effects of lysophosphatidylcholine in experimental sepsis. Nature medicine 2004;10:161-167.

[10] Phillips GB, Dodge JT. Composition of phospholipids and of phospholipid fatty acids of human plasma. J Lipid Res 1967;8:676-681.

[11] Campos AM, Maciel E, Moreira AS, Sousa B, Melo T, Domingues P, et al. Lipidomics of Mesenchymal Stromal Cells: Understanding the Adaptation of Phospholipid Profile in Response to Pro-Inflammatory Cytokines. Journal of cellular physiology 2015.

[12] Smani Y, Dominguez-Herrera J, Ibanez-Martinez J, Pachon J. Therapeutic efficacy of lysophosphatidylcholine in severe infections caused by Acinetobacter baumannii. Antimicrobial agents and chemotherapy 2015;59:3920-3924.

[13] Adebayo D, Morabito V, Andreola F, Pieri G, Luong TV, Dhillon A, et al. Mechanism of cell death in acute-on-chronic liver failure: a clinico-pathologic-biomarker study. Liver Int 2015.

[14] Fukiya S, Arata M, Kawashima H, Yoshida D, Kaneko M, Minamida K, et al. Conversion of cholic acid and chenodeoxycholic acid into their 7-oxo derivatives by Bacteroides intestinalis AM-1 isolated from human feces. FEMS microbiology letters 2009;293:263-270.

[15] Balog J, Sasi-Szabo L, Kinross J, Lewis MR, Muirhead LJ, Veselkov K, et al. Intraoperative tissue identification using rapid evaporative ionization mass spectrometry. Science translational medicine 2013;5:194ra193.
